# Elements of Viral Outbreak Preparedness: Lessons, Strategies, and Future Directions

**DOI:** 10.3390/v18010050

**Published:** 2025-12-29

**Authors:** Ibrahim Ahmed Hamza, Kang Mao, Chen Gao, Hazem Hamza, Hua Zhang

**Affiliations:** 1 Department of Water Pollution Research, National Research Centre, Giza 12622, Egypt; ia.ewess@nrc.sci.eg (I.A.H.); hazem.hamzanrc@gmail.com (H.H.); 2 State Key Laboratory of Environmental Geochemistry, Institute of Geochemistry, Chinese Academy of Sciences, Guiyang 550081, China; gchen05@163.com

**Keywords:** viral outbreak preparedness, outbreak response, emerging viruses, diagnostics, surveillance, one health, therapeutics

## Abstract

Emerging and re-emerging viruses continue to pose major threats to public health. Their ability to adapt, cross species barriers, and spread rapidly can trigger severe outbreaks or even pandemics. Strengthening preparedness with comprehensive and efficient strategies is therefore essential. Here, we explore the key components of viral outbreak preparedness, including surveillance systems, diagnostic capacity, prevention and control measures, non-pharmaceutical interventions, antiviral therapeutics, and research and development. We emphasize the increasing importance of genomic surveillance, wastewater-based surveillance, real-time data sharing, and the One Health approach to better anticipate zoonotic spillovers. Current challenges and future directions are also discussed. Effective preparedness requires transparent risk communication and equitable access to diagnostics, vaccines, and therapeutics. The COVID-19 pandemic highlighted both the promise of next-generation vaccine platforms and the necessity of maintaining diagnostic capacity, as early testing delays hindered containment efforts. Countries adopted various non-pharmaceutical interventions: risk communication and social distancing proved to be the most effective, while combined workplace infection-prevention measures outperformed single strategies. These experiences highlight the importance of early detection, rapid response, and multisectoral collaboration in mitigating the impact of viral outbreaks. By applying best practices and lessons learned from recent events, global health systems can strengthen resilience and improve readiness for future viral threats.

## 1. Introduction

The emergence of viral pathogens has led to several outbreaks worldwide during the last 20 years. Significant examples include the severe acute respiratory syndrome coronavirus (SARS-CoV) outbreak in China during 2002–2003 [1], the pandemic influenza A/H1N1 in 2009 [2], the Middle East respiratory syndrome (MERS) coronavirus that emerged in 2012 [3], the West Africa Ebola virus epidemic from 2013–2016 [4] and the Democratic Republic of the Congo (DRC) from 2018–2020, the Zika virus epidemic from 2016 to 2017 [5], the Mpox outbreak in 2022 and 2024 and the COVID-19 pandemic that originated in China in late 2019 [6]. Collectively, these outbreaks have provided recurring yet often underutilized lessons for pandemic preparedness. Table 1 summarizes selected major viral epidemics since 2009, their geographic contexts, and key preparedness lessons that inform the priorities discussed in this review.

Zoonotic spillover accounts for ~70–80% of newly emerging infectious diseases. Transmission pathways of zoonotic viruses are diverse, including foodborne or waterborne routes, vectors such as insects or other animals, and through contact, either directly with infected animals or indirectly with contaminated surfaces (fomites). The emergence and re-emergence of these threats reflect a complex interplay of viral, human, and environmental factors.

Viral factors, notably high mutation rates, recombination, and reassortment, facilitate rapid adaptation and success of emerging and re-emerging infectious diseases. For instance, RNA viruses lack proofreading activity in their RNA-dependent RNA polymerases (RdRp), resulting in a significantly elevated mutational rate of 10^3^–10^5^ substitutions per site per replication cycle, which promotes rapid genetic diversity and adaptation to new hosts [23]. In influenza virus, accumulating point mutations, in particular within the major surface protein, hemagglutinin (HA), constantly generate a multitude of genetic and antigenic variants [24]. Recombination and reassortment further enhance this inherent property, facilitating genomic modification and the emergence of new variants. Examples include poliovirus (PV) recombination that eliminates deleterious mutations, SARS-CoV-2 spike protein modifications through recombination, and influenza reassortment events (1957 Asian H2N2, 1968 Hong Kong H3N2, 2009 swine H1N1) that produced pandemic strains with significant global health consequences [25]. Collectively, these mechanisms result in viral quasispecies, which are diverse, dynamic groups that enhance virus adaptability under immune and environmental pressures [26].

Human factors, including globalization and urbanization, have accelerated viral spread through increased human mobility and density [27]. Air travel enables the rapid dissemination of viral pathogens across continents within 48 h, as demonstrated by the 2022 and 2024 worldwide Mpox outbreaks linked to international travel [28]. The expansion of urban areas into wildlife habitats results in increased human–animal contact. In more densely populated areas with reduced biodiversity, pathogen amplification increases as competent reservoir species become more abundant, as observed in the urban resurgence of dengue [27,29]. One behavioral factor that has hindered progress against vaccine-preventable diseases is vaccine hesitancy; in 2023, measles infections worldwide increased to 10.3 million, while vaccination coverage fell to 74% for second doses [30]. Conflict and humanitarian crises, such as those in Gaza, contributed to the PV resurgence in 2024, following a decline in vaccination rates from 99% in 2022 to 89% in 2023, which was attributed to a war-induced collapse of healthcare systems [31,32].

Environmental and ecological dynamics factors, such as deforestation and climate change, alter zoonotic risk landscapes. Habitat fragmentation forces wildlife into closer contact with the human population, leading to spillover events, as evidenced by outbreaks of Nipah virus linked to habitat destruction and bat displacement. Climate change also amplifies these risks further by altering the geographic distribution and abundance of vectors, such as mosquitoes and ticks. Given the rising temperatures, the expansion of *Aedes aegypti* habitats has driven dengue transmission to a record 13 million cases in 2024, a 361% increase over the 5-year cases, reflecting a long-term growth rate of 13% annually since 2000 [33].

**Figure 1:**
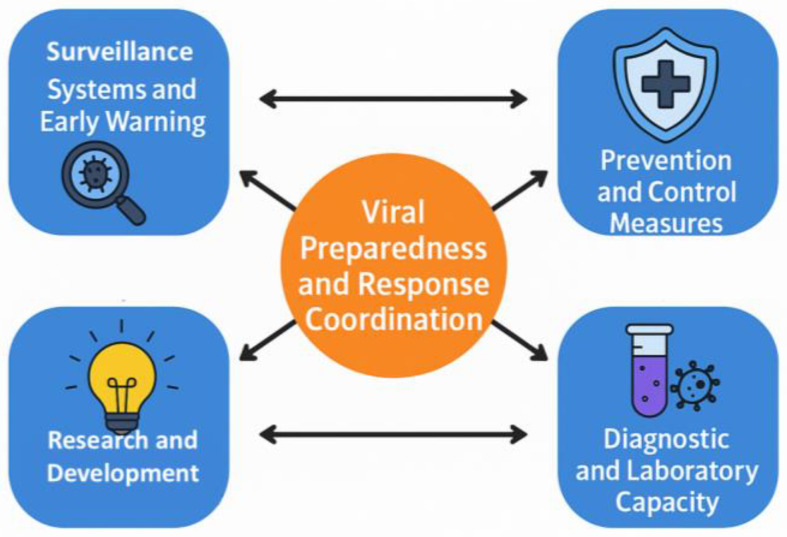
Conceptual framework for viral outbreak preparedness and response. The central hub represents coordination across four interdependent pillars: (i) surveillance and early-warning systems, including event-based, genomic, and environmental monitoring; (ii) diagnostic and laboratory capacity for rapid detection and characterization of viral threats; (iii) prevention and control measures such as vaccination, antivirals, and non-pharmaceutical interventions; and (iv) research and development supporting innovation in vaccines, therapeutics, and risk-assessment tools. This framework illustrates how integrated functions enable early detection, timely response, and overall system resilience.

In 2018, the World Health Organization (WHO) introduced the term “Disease X” to highlight the need for preparedness against unknown pathogens [34]. This concept was applied during the COVID-19 pandemic, when a novel betacoronavirus (SARS-CoV-2) showed that, despite advances in genomic surveillance, zoonotic spillover events could be unpredictable. Pathogen X has the potential to emerge, causing a future disease capable of causing epidemics or pandemics; therefore, there is a need to study the preparedness to develop a successful response and solutions. Moreover, in July 2024, the WHO updated the list of priority pathogens, focusing on entire families of bacteria and viruses rather than just specific entities to recognize that low-risk groups could evolve into high-risk ones. In this context, Pathogen X/Disease X refers to potential hazards that have not yet been identified. The update further incorporates “Prototype Pathogens,” which are viruses from high-risk families that will accelerate the development of vaccinations and diagnostics. Consequently, this review discusses in detail the key components of the outbreak preparedness plan, as illustrated in Figure 1 [35]. These integrated elements strengthen the ability to identify and respond to potential outbreaks before they escalate into widespread health crises.

Command and coordination optimize resource allocation and response organization. Robust and effective surveillance and early warning systems facilitate the rapid identification of emerging pathogens. Laboratory services are important for diagnostic confirmation and epidemiological studies. Prevention and control measures, including vaccination programs and public health communication, help contain outbreaks and protect vulnerable populations. Research and innovation continually facilitate the development of new treatments, diagnostic tools, and preventive measures to maintain readiness against emerging pathogens. Investment in these preparedness measures enhances public health protection and prevents future outbreaks from causing widespread devastation.

This review highlights current evidence and best practices for viral outbreak preparedness, with three main objectives: (1) to describe the key components of effective preparedness systems, including surveillance, diagnostics, prevention and control measures, research and development and response coordination; (2) to reflect on lessons learned from recent outbreaks, particularly COVID-19, to strengthen preparedness strategies; and (3) to identify gaps and future priorities in global health security. It targets public health officials, healthcare administrators, researchers, educators, policymakers, international organizations, and frontline healthcare workers involved in outbreak response. The review provides evidence-based insights to guide decision-making and support resilient health systems capable of responding effectively to future viral threats.

Several strategic frameworks for emergency preparedness have been published, including the WHO Strategic Framework for Emergency Preparedness [36], national guidance such as the U.S. Federal Emergency Management Agency’s incident management systems (U.S. FEMA, 2022), and international pandemic frameworks such as the WHO Pandemic Agreement [37]. These frameworks describe preparedness as a continuous cycle of anticipating hazards, mitigating impacts, responding to events, and recovering from consequences. This review does not aim to reproduce a comprehensive preparedness framework. Instead, it provides a standalone, evidence-based synthesis of viral outbreak preparedness, drawing on lessons from COVID-19, Mpox, and other viral threats. By focusing on key capacities—surveillance (including genomic and wastewater monitoring), diagnostics, vaccination and therapeutics, infection prevention and control, and research—this review provides an updated virology-specific perspective to inform national and global preparedness planning.

## 2. Methods

This narrative review synthesizes current evidence and best practices for viral outbreak preparedness. While it does not follow systematic frameworks such as PRISMA, a structured search was conducted across PubMed, Web of Science, Scopus, and key public health sources including WHO and Centers for Disease Control and Prevention (CDC) using keywords such as “viral preparedness,” “emerging viruses,” “genomic surveillance,” “wastewater surveillance,” “diagnostics,” “pandemic response”, “therapeutics” and “One Health.” The review prioritizes literature from 2015–2025 to reflect contemporary advances in viral preparedness, while older references are included selectively to provide historical context and a foundational understanding. Sources were evaluated for relevance, quality, and contribution to the review themes, and synthesized qualitatively to present current trends, challenges, and future directions.

## 3. Surveillance Systems and Early Warning

Effective viral surveillance is the cornerstone of pandemic preparedness, enabling early detection and tracking of novel threats before widespread transmission occurs. Systematic monitoring of high-risk regions and populations facilitates timely identification of emergent pathogens and zoonotic infections, supporting rapid risk assessment and preventive planning.

Beyond early warning, viral surveillance also plays a critical role in confirming and characterizing outbreaks by identifying clusters of illness or unusual disease patterns. Given the various modes of virus transmission and some viruses being zoonotic, an integrated surveillance system (One Health) is necessary, which includes the data from human, animal, and environmental sources to monitor and mitigate health risks at the interface of the human–animal environment. This approach helps in understanding disease transmission dynamics and supports coordinated response efforts.

International non-governmental organizations have developed several frameworks to address zoonotic diseases within the One Health context [38,39,40,41]. While the recent pandemics highlighted the broader applications of One Health, environmental monitoring of vector-borne viruses exemplifies its role in improving public health [42]. For example, in 2025, chikungunya cases reached approximately 240,000 worldwide, transforming from a regional concern to a global public health emergency.

While climate change and urbanization have expanded the habitat of *Aedes aegypti* and increased transmission potential, global spread has been facilitated by human mobility and international travel, leading to the emergence of major transmission hubs across multiple continents [43]. The Indian Ocean islands, France, and Italy have been particularly affected [44]. China has reported over 10,000 cases since July 2025, marking its largest recorded chikungunya outbreak, compared with only 519 cases between 2010 and 2019 [45].

### 3.1. Traditional Surveillance

#### 3.1.1. Passive Surveillance: Routine Monitoring and Systemic Gaps

Passive surveillance remains the cornerstone of routine public health monitoring. It depends on healthcare providers to voluntarily report notifiable diseases without systematic outreach [46]. Although this approach is cost-effective and scalable, it encounters considerable limitations due to systematic underreporting, which arises from variations in healthcare access, diagnostic practices, and healthcare-seeking behavior. The limitations of passive surveillance systems are well-documented [47].

Despite these limitations, passive systems are essential for establishing baseline epidemiological trends. In the United States, influenza-like illness (ILI) is routinely monitored across more than 120,000 healthcare providers before the pandemic, providing critical early warning data [48]. Additionally, the National Respiratory and Enteric Virus Surveillance System (NREVSS) in the United States demonstrates effective passive surveillance by collecting laboratory data on viruses such as the influenza virus and respiratory syncytial virus (RSV) from clinical laboratories nationwide (CDC, 2019). This structured plan provides comprehensive population surveillance and pathogen-specific verification, which forms the basis for outbreak preparedness.

#### 3.1.2. Active Surveillance: Targeted Case-Finding and Enhanced Sensitivity

Active surveillance addresses the limitations of passive systems by employing structured, proactive case-finding strategies through engagement with healthcare providers, contact tracing, and establishing specialized testing sites or field teams. The approach can capture underreported or asymptomatic cases and generate more detailed data. In Germany, studies have shown that active surveillance identified 27% more SARS-CoV-2 infections compared to passive reporting systems alone [49,50]. Community-based house-to-house surveys identified cases overlooked by passive approaches, leading to the interruption of transmission chains and targeted interventions, demonstrating how active surveillance can complement routine reporting systems.

Although active surveillance is more sensitive, it is labor-intensive, expensive, and often requires trained personnel, laboratory capacity, and logistical coordination, making it hard to scale up, especially in low-resource settings where infrastructure gaps persist. Global surveillance networks exemplify the successful implementation of active surveillance. The Global Influenza Surveillance and Response System (GISRS) combines virological and epidemiological data from over 130 countries, enabling real-time tracking of influenza variants and informing annual vaccine formulations. GISRS expanded during COVID-19 to monitor SARS-CoV-2 and RSV, demonstrating its potential for tracking other emerging threats. In addition, PV eradication relies on active surveillance for acute flaccid paralysis (AFP), with the WHO African Region conducting weekly case searches at priority health facilities to reduce the risk of undetected PV transmission in high-risk areas [51].

#### 3.1.3. Sentinel Surveillance: High-Resolution Pathogen Monitoring

Sentinel surveillance collects detailed epidemiological and virological data from a designated network of clinics, hospitals, or laboratories that serve as representative sites. It enables the targeted monitoring of specific viruses or demographic groups, providing high-resolution information while maintaining operational feasibility. However, data may not be fully representative of broader populations, especially in underserved or rural regions.

The adaptation of the influenza sentinel network during the COVID-19 pandemic enabled the detection of SARS-CoV-2 in 7349 severe acute respiratory illness cases in Kenya, with correlation to national outbreak trends (Pearson r = 0.58) [52]. This correlation indicates that influenza surveillance networks can be helpful for early detection of new respiratory pathogens. However, the moderate strength of the relationship suggests that additional surveillance approaches are needed to fully track outbreaks. These findings in Kenya align with broader evidence that sentinel surveillance systems can serve as early warning tools for emerging pathogens by capturing shifts in pathogen circulation before they are reflected in national case-based reporting.

For instance, the European Respiratory Virus Surveillance Summary (ERVISS) collects standardized sentinel data from 38 EU countries to monitor respiratory viruses such as influenza, RSV, and SARS-CoV-2 in real time (European Centre for Disease Prevention and Control) [53]. Similarly, the sentinel Enhanced Dengue Surveillance System (SEDSS) identified Zika virus circulation eight weeks earlier than passive systems in Puerto Rico, demonstrating its early warning capabilities. Nevertheless, sentinel systems may introduce geographic bias, as urban or better-resourced areas are often overrepresented [54].

The integration of genomic surveillance efforts through systems such as NREVSS and GISRS also contributes to genomic surveillance efforts and is discussed further in the Genomic Surveillance section. While traditional surveillance is widely used, approaches such as genomic surveillance, digital surveillance, and wastewater-based surveillance (WBS) are indeed considered among the most cutting-edge and innovative approaches in the field of public health surveillance; therefore, they will be covered in detail in the following sections.

#### 3.1.4. Examples of National Surveillance Systems

While general surveillance frameworks provide the foundation for outbreak monitoring, national systems demonstrate how structural and organizational differences influence effectiveness, responsiveness, and integration with global networks. The following examples from the European Union, the United States, and China highlight contrasting approaches to centralization, data sharing, and multi-level coordination, illustrating the trade-offs inherent in national surveillance design and providing lessons for building more effective and responsive preparedness systems.

European Union Surveillance Systems:

The European Surveillance System (TESSy) serves as the central data repository for communicable disease surveillance, receiving standardized data from national public health authorities [55]. TESSy collects case-based data on diseases of public health importance, including mandatory reportable diseases (e.g., measles, pertussis, meningococcal disease, COVID-19) and optional surveillance targets.

Integration with the EU Early Warning and Response System (EWRS) enables rapid communication and mandatory reporting of suspected public health emergencies of international concern. Laboratory surveillance is coordinated through the European Reference Laboratory network and the European Surveillance System for Special Pathogens (ESSPnet), enabling rapid confirmation of suspected cases and tracking of laboratory-confirmed disease trends. During the COVID-19 pandemic, EU surveillance demonstrated both strengths (rapid identification of variants through TESSy data and real-time reporting) and weaknesses (initial delays in harmonizing case definitions across member states, variable testing capacity among countries). Taken together, these multi-level surveillance mechanisms highlight the challenges of harmonizing public health data across multiple member states, where centralized TESSy coordination must interact with heterogeneous national reporting systems.

United States Surveillance Systems:

The United States employs a decentralized surveillance system that involves the CDC, state health departments, and local jurisdictions. The National Notifiable Diseases Surveillance System (NNDSS) is the primary passive system [56]. Thousands of jurisdictions report notifiable diseases to state health departments, which then send the information to the CDC through the National Electronic Disease Surveillance System (NEDSS). Active surveillance for certain pathogens is performed by sentinel programs, which include networks of labs and clinics. For example, FoodNet conducts surveillance for foodborne diseases, while influenza is monitored through systems such as ILINet and FluSurv-NET. Event-based surveillance relies on the CDC’s Emergency Operations Center and international partnerships. During the COVID-19 pandemic, genomic surveillance grew quickly. The CDC’s SPHERES (SARS-CoV-2 Sequencing for Public Health Emergency Response, Epidemiology, and Surveillance) initiative brought sequencing data together from across the country [57]. However, decentralization creates variable reporting times and incomplete federal data aggregation, illustrating tensions between centralized coordination and decentralized implementation.

China’s Surveillance Systems:

China operates a centralized infectious disease surveillance system led by the National Health Commission and implemented through the China Notifiable Disease Surveillance System (CNDSS/CNOTC). Launched in 2004, CNOTC is a web-based platform requiring rapid, standardized reporting of notifiable diseases nationwide, enabling national trend analysis and outbreak detection by the China CDC [58,59]. Laboratory surveillance includes the National Influenza Surveillance Network, with about 400 sentinel sites collecting weekly respiratory specimens. Recent innovations involve WBS for early detection of SARS-CoV-2. While the centralized approach enables swift policy responses, concerns persist regarding data accuracy, potential underreporting, and limited real-time transparency [59]. China has improved data sharing with international platforms, though occasional delays in outbreak reporting remain. Overall, China’s centralized system demonstrates the advantages of rapid reporting and policy response, and also illustrates trade-offs between centralized control and data transparency, highlighting the importance of accuracy and international data sharing for global surveillance.

EU, U.S., and Chinese surveillance systems demonstrate that centralized coordination accelerates reporting and policy action, decentralization enhances local responsiveness, and integrating genomic, digital, and environmental data is critical for robust, timely outbreak detection and global preparedness.

### 3.2. Genomic Surveillance

Genomic surveillance systematically tracks the viral genetic evolution to understand transmission dynamics, adaptation, and impact on public health. Enabled by rapid Next-Generation Sequencing (NGS) technologies and portable sequencing platforms, genomic surveillance has transformed outbreak response in both resource-rich and resource-limited settings.

Genomic surveillance transformed Ebola outbreak responses, particularly in resource-limited regions. During the 2018–2020 outbreak in the DRC, an end-to-end genomic surveillance system sequenced 17% of confirmed cases, revealing superspreading events and guiding ring vaccination strategies [16]. Portable technologies like the Oxford Nanopore MinION platform enabled real-time sequencing in field laboratories, reducing turnaround times to <48 h, demonstrating proof-of-concept for decentralized, rapid genomic workflows. In 2025, Ugandan scientists built on these advances to confirm a Sudan Ebola virus outbreak within 24 h using decentralized sequencing and phylogenetic analysis, demonstrating scalability in austere environments [60].

The COVID-19 pandemic highlighted the critical role of genomic surveillance in public health response. Through continuous sequencing, scientists have found mutations associated with changes in virulence, transmission dynamics, host range, immune escape, and therapeutic resistance [61,62]. The COVID-19 Genomics Consortium (COG-UK) in the UK was able to sequence more than 20% of confirmed cases throughout the country. This major project provided not only genetic information but also valuable data, combining sequencing findings with epidemiological trends to map and interrupt the transmission chain in near real time [63].

Similarly, during the monkeypox (MPXV) outbreak, rapid sequencing identified the virus and revealed about 20 times accelerated mutation rates compared to its animal host reservoir [64]. This finding highlights the importance of integrating genomics with epidemiological surveillance, maintaining sequencing capacity and multisectoral collaboration.

Recognizing these advantages, the WHO developed a long-term plan for genetic monitoring. Their 2022–2032 strategy aims to integrate sequencing capabilities into national public health systems. This includes linking them to existing surveillance platforms, building sequencing capacity, and fostering collaboration among stakeholders [64]. This represents a fundamental shift from reactive to proactive genomic surveillance in pandemic preparedness.

Several models demonstrate successful implementation of global data sharing initiatives. The Global Initiative on Sharing All Influenza Data (GISAID) and the GISRS are two examples of platforms that are important for worldwide cooperation. GISRS, a WHO-coordinated network founded in 1952, currently includes laboratories from 149 countries. It connects virological and epidemiological data for real-time tracking of influenza variants, and annual vaccine selection [64]. These methods provide a robust framework for establishing genetic surveillance for other diseases.

By early 2024, GISAID had shared more than 14 million SARS-CoV-2 genomes, making it a real-time worldwide archive that was necessary to accelerate vaccine research and variant risk assessment [65]. WHO Global Genome Surveillance Strategy (2022–2032) aims to ensure that all 194 member nations have genome sequencing capabilities by 2032. This directly addresses the equity gaps that were exposed during the COVID-19 pandemic [35]. By 2022, networks like PAHO’s COVID-19 Genomic Surveillance Network had sequenced and exchanged more than 307,000 genomes throughout Latin America and the Caribbean, facilitating tailored public health interventions [66].

Genomic surveillance proved essential in tracking the exceptional 2024 H5N1 avian influenza epidemic in U.S. dairy calves, revealing that it began from a single wild bird importation, followed by reassortment with avian influenza viruses, generating the B3.13 genotype [67]. Whole-genome sequencing identified PB2 mutations facilitating mammalian adaptation, though no traditional HA mutations supporting human adaptation were detected [68]. The ongoing zoonotic risk, especially for occupationally exposed dairy workers, underscores the value of genomic monitoring for real-time outbreak management. Bulk milk testing for herd-level detection and real-time monitoring of viral transmission, informed by cow movement patterns, exemplifies the operational integration of genomic surveillance in One Health frameworks [67,69].

Although genomic surveillance holds promising potential, it encounters several challenges, including technical, logistical, analytical, and equity dimensions. Genomic surveillance demands substantial investment in sequencing infrastructure, reagent supply chains, specimen transport, and data systems integration; requirements that strain resource-limited laboratories. Also, false-negative sequencing results can arise from suboptimal viral loads, late-stage specimen collection, or variant-induced primer/probe mismatches [70].

Genomic surveillance logistics are even more demanding, including the timely transport of clinical specimens, maintaining high-throughput sequencing capacity, ensuring supply-chain stability for reagents and flow cells, and integrating data upload pipelines into national reporting systems. Many countries experienced substantial sequencing slowdowns during COVID-19 surges due to reagent shortages, staff burnout, or overwhelmed laboratory networks [70].

Bioinformatic infrastructure gaps severely limit genomic data utility. High-quality interpretation requires robust pipelines for genome assembly, variant calling, phylogenetic analysis, and metadata management—tools that are unevenly available across regions [71]. Limited computational infrastructure, inconsistent quality-control standards, and shortages of trained bioinformaticians delay data processing and reduce the comparability of genomic datasets [72]. The Proliferation of national pipelines fragments data interoperability, and incomplete or missing clinical-epidemiological metadata often hinders real-time analysis [73].

Country-level capability differences amplify disparities in outbreak detection equity. High-income countries often maintain extensive sequencing networks, stable funding, strong public health laboratories, and integrated data systems. Conversely, many low- and middle-income countries (LMICs) rely on external partners for sequencing, experience long turnaround times, and lack sustained financing for surveillance infrastructure, and remain under-represented in global databases like GISAID [72,74]. These gaps delay variant detection, skew global genomic databases toward high-income nations, and undermine equitable pandemic preparedness.

These logistical and analytical barriers manifest differently depending on pathogen characteristics and outbreak context. Ebola outbreaks in remote areas face acute specimen transport challenges, while global influenza surveillance requires processing high sample volumes across hemispheres [75]. Emerging respiratory viruses like SARS-CoV-2 require rapid turnaround and extensive sequencing infrastructure, while vector-borne diseases may tolerate longer analytical windows. Consequently, pandemic preparedness requires pathogen-specific genomic strategies, sustained capacity investment, and ethical data-sharing frameworks ensuring timely, reliable outbreak intelligence.

### 3.3. Digital Surveillance

Digital surveillance offers a cost-efficient and time-effective alternative to traditional monitoring approaches. However, the data obtained can be noisy, making it challenging to acquire reliable and precise information compared to conventional methods. One of the pioneering entrants in digital surveillance is the Program for Monitoring Emerging Diseases (ProMED), which gathers information from diverse sources, including media reports, official reports, social media, local observers, and a global network of clinicians [76]. ProMED has been instrumental in raising prompt alerts about major outbreaks, such as the Zika virus spread to the Americas and the early detection of SARS [77,78]. It gained international recognition for its thorough report and risk assessment of unusual pneumonia cases in Wuhan, China, in December 2019. HealthMap also operates alongside other prominent tools for monitoring disease outbreaks. HealthMap utilizes various sources, including news aggregators, firsthand accounts, and official and unofficial channels, to visualize alerts on interactive maps. HealthMap and ProMED have provided surveillance data to the Epidemic Intelligence from Open Sources (EIOS) system developed by the WHO [79].

Additionally, modern digital systems utilize artificial intelligence and large-scale data mining to detect outbreaks earlier than traditional surveillance methods. EPIWATCH, an AI-powered epidemic intelligence system that tracks early signs of infectious disease outbreaks by searching open-source data, like news, social media, and government reports, in more than 40 languages. The system demonstrated an 88% accuracy in detecting outbreak signals. EPIWATCH reported 65 mumps outbreaks worldwide, often before WHO or CDC notifications, and it also reported early signs of Ebola and COVID-19 [80,81]. The platform is supplemented by tools such as EpiRisk, which swiftly evaluates the risk and possible severity of outbreaks using country- and pathogen-specific data, and FluCast, which predicts the severity of coming influenza seasons in real time to aid in preparedness and rapid response [82].

Other systems, such as the Global Public Health Intelligence Network (GPHIN), FluTracking, and BioSense 2.0 platforms, demonstrate the growing importance of digital surveillance in enhancing traditional disease surveillance efforts and facilitating rapid responses to emerging infectious disease threats [83]. Canada’s GPHIN, founded in 1997, employs natural language processing and machine learning to scan news, social media, and other digital sources in nine languages [84]. GPHIN enhances WHO epidemiological intelligence by approximately 20% through the filtration and prioritization of data related to public health crises. During the 2009 H1N1 pandemic, GPHIN found unusual respiratory cases in Mexico weeks before the official reports, facilitating prompt international collaboration. Nevertheless, its 2019 restructuring, which focused on local threats, temporarily reduced its global impact, highlighting the ongoing challenges of balancing resource allocation with international health security obligations.

FluTracking is an online Australian health surveillance system, launched in 2006 to detect the potential spread of influenza. It uses weekly online surveys to collect data on symptoms of influenza-like illness, vaccination status, and healthcare-seeking behaviors. With approximately 150,000 individuals in Australia and New Zealand, it provides insights into community-level transmission patterns and vaccine efficacy. Throughout the COVID-19 pandemic, FluTracking expanded its scope to include monitoring SARS-CoV-2 symptoms, showing that 25% of mild cases did not pursue testing. The efficiency of the platform is attributed to minimal user effort (10-second surveys) coupled with real-time analytics, which directly guide public health responses.

The BioSense 2.0 platform in the United States has been integrated into the CDC National Syndromic Surveillance Program (NSSP), which collects electronic health information from participating emergency departments (EDs) nationwide to detect abnormalities in symptom patterns [84]. The NSSP, overseen by the CDC, employs the BioSense Platform, which has technologies such as the Electronic Surveillance System for the Early Notification of Community-Based Epidemics (ESSENCE). The approach enabled the finding of localized COVID-19 clusters in early 2020 via evaluating emergency department visits with near-real-time latency, generally within 24 h, to augment conventional monitoring efforts. However, program efficacy is hampered by limitations in data exchange and system compatibility.

Moreover, several mobile apps, including Outbreaks Near Me (formerly Flu Near You), CoronaData (Robert Koch Inst), and SickWeather (US), can contribute to digital viral outbreak surveillance. Outbreaks Near Me, is an online participatory surveillance instrument used to monitor influenza-like diseases in the United States and Canada. The platform’s incorporation of vaccine locator tools significantly improves public health functionality; yet, participation bias, such as the over-representation of health-conscious populations, persists as a drawback. The CoronaData app in Germany, managed by the Robert Koch Institute, collects signs, such as heart rate and sleep patterns, from fitness trackers to detect potential COVID-19 cases [84]. This application identified presymptomatic cases during the 2020–2021 waves by analyzing deviations from baseline health values, demonstrating the potential of wearable technology in passive surveillance. Moreover, SickWeather uses AI to scan illness-related keywords in social media posts to generate maps of outbreaks in real time. During the 2017–2018 flu season, it detected regional spikes in respiratory illnesses 7–10 days before the CDC did, showing how powerful digital chatter can be at predicting things. The app also combines user-reported symptoms with Bluetooth-enabled thermometer data to enhance the surveillance network.

On the other hand, critics warn against relying too heavily on social media data that has not been verified, which could spread misinformation. Despite the advantages of digital surveillance, challenges such as data quality and standardization, privacy risks, and equity gaps may limit its effectiveness.

### 3.4. Wastewater-Based Surveillance

Surveillance of viruses in sewage has emerged as a valuable approach for tracking human infections and the circulation of viruses. The WHO has incorporated sewage testing for PV into its global plan to eradicate PV, working alongside existing efforts to track cases of AFP surveillance. Noteworthy instances from Finland [85], Palestine [86], and The Netherlands [86] underscore the utility of sewage monitoring in revealing the widespread geographical circulation of the virus, detecting epidemics even in areas lacking reported cases of paralysis, or even prior to the onset of poliomyelitis cases. A notable example is PV surveillance in Egypt. Egypt has implemented a comprehensive PV surveillance system since 2000, including regular training for personnel and environmental monitoring of sewage. This successful system contributed to the country achieving polio-free status in 2006, through both surveillance for AFP by healthcare workers nationwide and environmental monitoring of sewage.

At the initial stage of the COVID-19 pandemic (March 2020), we highlighted the potential of WBS and early warning of infectious disease outbreaks, as well as for tracing the sources of COVID-19 [87]. Then, multiple studies across various countries successfully detected SARS-CoV-2 RNA in wastewater. These studies include Egypt [88], Canada [89], Italy [90], the Netherlands [91], Australia [92], Germany [93], Japan [94], the United Arab Emirates [95], and the United States [96,97]. In Amersfoort, the detection of SARS-CoV-2 RNA in wastewater six days before the first clinically confirmed cases illustrates how WBS can serve as an early-warning system. This lead time could enable public health authorities to implement targeted testing, contact tracing, and localized containment measures, potentially reducing transmission before outbreaks are recognized through conventional surveillance [91]. Ahmed et al. [92] observed varying SARS-CoV-2 RNA levels from three wastewater treatment plants in Australia, highlighting the importance of local health department collaboration for meaningful trend interpretation. Moreover, seven-week wastewater monitoring in France correlated SARS-CoV-2 levels with nationwide lockdown timing, demonstrating WBS utility for population-level outbreak tracking [98]. A study in Belgium found that wastewater sampling can help assess the community spread of respiratory viruses, such as influenza and respiratory syncytial virus [99]. This work illustrates how WBS functions as a complementary epidemiological tool to traditional clinical surveillance, providing real-time insights into subclinical and clinical virus circulation at the population level and thereby informing targeted public health interventions. A comprehensive study on WBS found that it is closely associated with COVID-19 cases in the majority of 107 US counties. These relationships were more pronounced in counties with a larger population and urban areas. In situations where routine COVID-19 surveillance data are less reliable, WBS may be employed to monitor local SARS-CoV-2 incidence trends [100].

Similarly, numerous laboratories worldwide have monitored MPXV DNA in wastewater, exploring its potential utility as a management tool for MPXV. While recent studies have shown the feasibility of monitoring MPXV using WBS, the concept remains at a proof-of-concept stage for MPXV surveillance, as most of these investigations have indicated relatively lower levels of MPXV in wastewater compared with reported levels of SARS-CoV-2 elsewhere [101]. The low detection rate of MPXV in sewage warrants investigation to determine whether it reflects a lower incidence of MPXV clinical cases or MPXV is less abundant in wastewater. Several studies on the WBS of SARS-CoV-2 and MPXV are summarized in Table 2, encompassing diverse sampling matrices, concentration methods, and detection performances.

Despite their growing importance, both WBS and genomic surveillance face inherent limitations that can compromise early detection. False negatives in WBS may arise from low viral shedding at the population level, dilution effects during heavy rainfall, chemical degradation of viral nucleic acids in sewer systems, and variability in sampling frequency or catchment size [102]. These limitations underscore the need for multipronged surveillance architectures rather than reliance on any single modality. However, data interpretation should be carefully discussed because sampling protocols such as grab, composite, traditional active, and passive sampling may influence the results [103]. Since WBS primarily relies on qPCR, PCR-inhibitory substances present in the wastewater samples may interfere with the qPCR, leading to an underestimation of the potential public health hazards of waterborne viral pathogens [104].

Beyond technical sensitivity, logistical challenges remain a significant barrier to scalable surveillance. WBS programs require standardized sampling protocols, temperature-controlled transport systems, and consistent laboratory turnaround times—conditions that are difficult to maintain during large outbreaks.

**Table 2:** Wastewater-Based Epidemiology Studies for SARS-CoV-2 and Monkeypox Virus (MPXV).

Study Location	Sample Type	Concentration Method	Detection Rate	SARS-CoV(-2) Titre	Key Findings	Reference
**SARS-CoV-2**						
New Haven, USA	Primary sewage sludge	Direct extraction by RNeasey PowerSoil Total RNA Kit	100% (10-week study)	3.23–5.66 log_10_ gc/mL	Viral RNA tracked clinical cases 6–8 days ahead; correlated with hospitalizations.	[105]
Murcia, Spain	WWTP influent	Aluminum hydroxide adsorption	84% (35/42) WWTPs	Avg (5.1–5.6 log_10_ gc/L)	Detected SARS-CoV-2 RNA 12–16 days before clinical confirmation in low-prevalence regions.	[106]
	Secondary treated		11%(2/18)	Avg(5.4 log_10_ gc/L)		
Porto, Portugal	Untreated/treated	PEG 8000 precipitation	81% (39/48) in untreated liquid samples, 0% in treated samples	0–0.15 gc/ng RNA	Wastewater-based surveillance to complement clinical testing	[107]
Milan, Rome, Italy	WWTP influent	a two-phase (PEG-dextran) separation	50% (6/12 samples)		The virus was found in Italian wastewaters, even before the first reported clinical case in the country	[90]
Finland	municipal wastewater influent	ultrafiltration	79%	6.62–8.72 log_10_ gc/day/person	WBS tracks SARS-CoV-2 trends at the community level	[108]
Madrid, Spain	Network-wide sewage	Not reported	Not reported	0–7 log_10_ gc/L	Strong correlation (3–8-day lead) with hospitalizations; identified regional transmission waves.	[109]
Japan	Weekly/biweekly influent	PEG 8000	46.7% (21/45(Omicron wave)	4.08–4.54 log_10_ gc/L	SARS-CoV-2 in wastewater increased when the number of confirmed cases exceeded 10 per 100,000 people.	[94]
Japan	Weekly influent wastewater	PEG 8000	67% (88 out of 132)	3.5–6.3 log_10_ gc/L	Significantly higher SARS-CoV-2 RNA concentrations were detected during the Omicron variant phase	[110]
Northern California USA	Wastewater settled solids	-	100% (974/974)	6.57 log_10_ gc/g dry weight	Both grab and composite samples performed similarly for SARS-CoV-2 quantification in settled solid	[111]
Egypt	WWTP influent	PEG 6000 precipitation	62.5% (30/48)	-	Highlighted value for LMICs surveillance.	[88]
Canada	Primary clarified sludge, post-grit solids	PEG-8000 PPT	N1 gene: 92.7%, N2 gene 90.6% N1 gene 79.2%, N2 gene 82.3%	3.23–5.58 log_10_ gc/L	PMMoV normalization improved correlation with clinical data (r = 0.84).	[112]
Netherlands	WWTP influent	Ultracentrifugation	69% (for late-epidemic rounds, 100%)	3.4–4.3 log_10_ gc/L	Detected SARS-CoV-2 RNA 6 days before the first clinical cases in Amersfoort.	[91]
Australia	WWTP influent (3 plants)	Electronegative membrane filtration and ultrafiltration	N1 gene 30.1% E: 3.17% N2: 0%	3.05 log_10_ gc/L–5.08 log_10_ gc/L	Early detection, 3 weeks before the first clinical case; highlighted the need for public health collaboration.	[113]
Germany	WWTP influent and effluent	centrifugal ultrafiltration	77% (17/22 samples)	Inlet: 3.48–4.30 log_10_ gene equivalents/L Outlet: 3.43 to 4.57 log_10_ gene equivalents/L	The replication potential tests were negative for wastewater samples.	[93]
UAE	Municipal wastewater (Influents and effluents)	Ultrafiltration/PEG 8000 PPT	85% (untreated)	2.88 log_10_ gc/L–4.53 log_10_ gc/L	WBE can help authorities take prompt actions to contain a potential outbreak	[95]
USA (Montana)	Municipal wastewater(influent)	Centrifugal ultrafiltration	53% (9/17)	2.85–4.12 log_10_ gc/L	Viral titers correlated with case numbers; provided an early warning.	[97]
USA (Massachusetts)	Municipal wastewater	PEG precipitation	100% (all samples)	1.76– 2.48 log_10_ gc/mL	Viral titers anticipated clinical trends by 4–10 days.	[96]
France	WWTP influent (3 plants)	Centrifugation	100%	4.70–6.48 log_10_ gc/L	Viral RNA trends mirrored the national lockdown and resurgence.	[98]
Veneto, Italy	Raw wastewater	PEG precipitation.	High correlation with clinical cases	0–2.85 log_10_ gc/µL	Wastewater peaks preceded clinical cases by 5.2 days; CUSUM charts are effective for early outbreak detection	[114]
Chengdu, China	A composite Wastewater treatment plant influent	PEG precipitation	0.012–3.27%	0.21–1.62 log_10_ gc/mL	Model predicted infections using viral load and population size; provided early warning for FISU Games.	[115]
Arkansas, USA	Wastewater treatment plant samples	filtered and eluted to approximately 500 µL using a column-based system		>1 log_10_ gc/mL–6 log_10_ gc/mL	Amplicon sequencing tracked variants effectively; S-gene detection was lost with JN.1 predominance.	[116]
**Denmark**	Influent wastewater	NanoTrap Microbiome A particles	consistent detection (LoD: 4 copies/reaction for N1, 2 for N2)		Including wastewater SARS-CoV-2 levels in models improved the prediction accuracy of COVID-19 hospital admissions up to 2 weeks in advance	[117]
**Monkeypox Virus**						
**Study Location**	**Sample Type**	**Concentration Method**	**Detection Rate**	**MPXV Titre**	**Key Findings**	**Reference**
Netherlands	Wastewater 24-h composite	Centrifugation	42% (45/108 samples)	Not quantified	The detection patterns in wastewater aligned with the confirmed monkeypox cases	[118]
Baltimore, USA	Grab, 24-h composite	PEG precipitation, adsorption-elution	72% (13/18 samples)	Not quantified	PEG precipitation is more effective than AE; no correlation between wastewater MPXV and clinical cases	[119]
Paris, France	24-h composite	Centrifugation	10.6% (34/321)	3.30−4.60 log_10_ gc/L	Strong correlation between MPXV concentration and weekly MPXV cases; early detection demonstrated	[120]
California, USA	24-h composite influent, solids	Nanotrap particles (liquid), buffer suspension (solids)	26.5 (76/287)	4.4 log_10_ gc/g (10^3^-fold higher in solids)	MPXV DNA is more concentrated in solids; positive correlation with reported cases	[121]
Miami, USA	Grab, Wastewater	Electronegative filtration	3.1% (1/32, hospital Wastewater) 38.5% (5/13 regional WWTP	3.8 log_10_ gc/L 4.0–4.42 log_10_ gc/L	First detection in July 2022; positivity increased during the study period; detected in hospital and municipal wastewater	[122]
Canada	24-h composite	Centrifugation	G2R_G: 16%, G2R_WA: 22%, G2R_NML: 76%	Not specified	In-house G2R_NML assay outperformed CDC assays for MPXV surveillance	[123]
Italy	Airport Wastewater 24-h composite	PEG/NaCl precipitation	15% (3/20)	Not quantified	Detection using N3R, F3L, CDC G2R_G; airport wastewater also analyzed	[124]
Thailand	Grab	Centricon Plus-70 ultrafilter	9.52% (6/63)	4.2−4.9 log_10_ gc/L	Feasibility demonstrated in Southeast Asia; positivity increased over the monitoring period	[125]
Spain	Grab	Aluminum adsorption-precipitation	18% (56/312 samples)	3.3−4.9 log_10_ gc/L	Large-scale study; aluminum-based concentration effective for MPXV detection	[126]
Multiple US States	24-h composite untreated	Vacuum filtration + pre-amplification	13% (8/60 samples)	Not quantified	Pre-amplification reduced false negatives by 87%; detected in multiple states; detection during case increases and waning.	[127]
Slovenia	24 h composite untreated Wastewater	affinity-based capture using Nanotrap particles	0% during the monitoring period	Not detected	No MPXV detected June–September 2023; validated methods for emergency response.	[128]
Zibo, China	Wastewater at high-risk sites	Magnetic beads, PEG, ultrafiltration	14.3 (1/7) Detected September 2023	3.1 log_10_ gc/mL	First MPXV detection in Chinese wastewater; NGS confirmed IIb branch C.1 lineage; suggested hidden transmission	[129]
United States	Composite/Grab Wastewater samples	Various methods	2.7% (95/3492)	Not specified	Sensitivity increased with case number; high predictive value; useful complement to case surveillance	[130]
USA	Composite	Adsorption extraction	3.8% (5/131)	3.2 log_10_ gc/L	Same-day result is feasible with the affinity capture method and microfluidic digital PCR	[131]
Poland	Composite	Not specified	20.5% (9/44)	Not specified	The MPXV virus detection does not correlate with the number of hospitalizations in Poznan, Poland	[132]
Korea, Seoul	Grab&composite	Dyna beads of the KingFisher equipment	1.2% (1/82)	Not specified	Wastewater-based surveillance is feasible for tracking low-prevalence, socially stigmatized pathogens at the community	[133]

While WBS and other surveillance tools provide valuable early warning, coverage remains fragmented and delayed, particularly in low-resource settings. Global health assessments have noted incomplete data reporting, limited laboratory capacity, and underutilized environmental and One Health data streams. Addressing these gaps requires expanding lab and field capacity, improving data sharing, and integrating human–animal surveillance networks (see Table 3).

### 3.5. A Coordinated International Effort and Data Sharing

The WHO established a global disease surveillance infrastructure through its International Health Regulations (IHR), adopted in 1951 and updated in 2005 to ensure coordinated outbreak response [137]. GISRS provides standardized communication protocols and reporting frameworks to prevent disease spread and address specific pandemic threats [12].

The emergence of COVID-19 prompted the WHO’s swift declaration of a Public Health Emergency of International Concern (PHEIC) and the implementation of a global surveillance system. This action was built upon lessons learned from previous outbreaks such as SARS (2003) and H1N1 (2009) [138], highlighting the crucial role of timely and extensive data for decision-making supported by evidence. Projects such as the Pandemic Influenza Preparedness (PIP) Framework, adopted in 2011, reinforce this principle by ensuring equitable access to vaccines through virus-sharing agreements. WHO employs standardized methods and continuously updates guidance to maintain data consistency across diverse sources.

The 2009 H1N1 pandemic demonstrated critical gaps in real-time data exchange, which led to the development of GISAID. By removing obstacles to pre-publication data access, GISAID, introduced in 2008, has transformed genomic data sharing and enabled real-time genomic surveillance of influenza [139]. Moreover, during the COVID-19 pandemic, GISAID facilitated the sharing of over 15 million SARS-CoV-2 sequences, as of mid-2023, hence aiding in the tracking of variants and informing vaccine development [64].

The WHO Health Emergencies Programme, Epidemic Intelligence from Open Sources (EIOS) system, launched in 2017, supplements these efforts by using AI to scan over 150,000 news and social media posts daily across more than 40 languages. Its evaluation in Africa found that EIOS detected 81% of public health events, often before the official reporting, with 47.4% sensitivity in identifying outbreaks like Ebola and COVID-19 before national notifications [135,140].

In addition, the Global Outbreak Alert and Response Network (GOARN, https://goarn.who.int) (accessed on 10 August 2025) mobilizes resources and experts during public health emergencies. It consists of more than 200 technical institutions and networks worldwide, by sending field epidemiologists, boosting laboratory networks, and improving risk communication. GOARN played an essential role during the 2018 Ebola outbreak in the DRC. Similarly, the CDC Global Disease Detection (GDD) Operations Center, a more specific program that plays a pivotal role in outbreak response, conducts event-based surveillance. It acts upon rumors and signals, even when governments hesitate to disclose information, utilizing media reports and other open-source data. For example, in 2017, media reports alerted the GDD center to a potential dengue outbreak in Egypt, enabling early action [141].

The GDD collaborative approach prioritizes information sharing through established platforms, fostering collaboration among stakeholders. This was exemplified during the 2018 Ebola outbreak in the DRC, where a shared data portal facilitated a coordinated response. The GDD swift response and ability to debunk misinformation were also demonstrated in 2014 during Ebola outbreak in Guinea and in 2022, smallpox rumors in Yemen [141].

## 4. Diagnostic and Laboratory Capacity

In crafting a comprehensive preparedness plan for viral outbreaks, diagnostics play a crucial role in rapidly detecting epidemic pathogens and guiding efficient public health interventions aimed at effective outbreak containment. Governments should ensure that readily accessible, cost-effective, and high-performing diagnostic tests are available on a large scale within weeks of identifying an emerging outbreak. This requires systematic investment in adaptable diagnostic platforms, robust laboratory infrastructure, and coordinated response frameworks, ensuring tests are rapid, sensitive, and deployable across various settings, including homes, Point-of-Care (POC) facilities, and central laboratories. These technologies should also be affordable and accessible to support national requirements for regular diagnostic testing, screening, and surveillance during prolonged periods of mass demand.

During major outbreaks, global supply chains quickly collapsed, causing widespread shortages of extraction kits, enzymes, lateral-flow membranes, cartridges, and even basic consumables [142]. These constraints were intensified by export bans, competition between countries, and dependence on a small number of manufacturers. Many POC assays also require cold-chain storage, making them difficult to use consistently in low-resource or remote settings where high temperatures degrade reagents and reduce test sensitivity [143]. Cartridge-based systems faced manufacturing bottlenecks that prevented them from meeting the demand required during large-scale surges [144].

Regulatory pathways, such as the Food and Drug Administration (FDA) Emergency Use Authorization (EUA) and the WHO’s Emergency Use Listing, accelerated the approval of new assays, allowing for the rapid expansion of testing early in the pandemic. However, differences in approval criteria across countries, occasional adoption of suboptimal tests, and variable regulatory capacity highlighted the need for coordinated global regulatory frameworks, shared validation standards, and stronger infrastructure in lower-resource settings [145].

**Figure 2:**
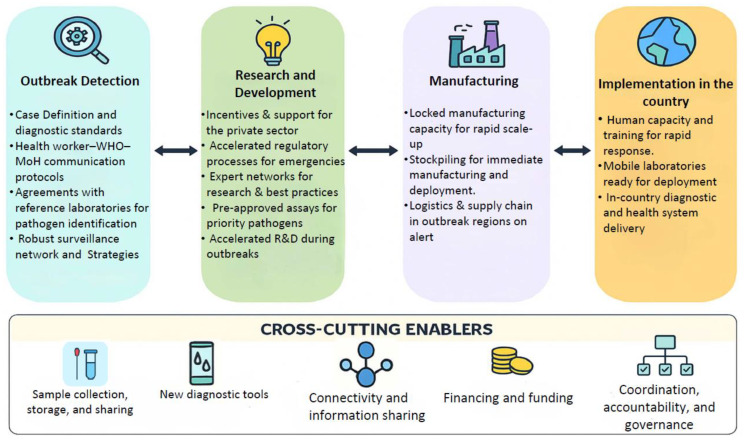
A framework for diagnostic preparedness. The figure illustrates the diagnostics preparedness value chain, highlighting phase-specific activities and five cross-cutting enablers that collectively strengthen rapid outbreak detection and response. Bidirectional arrows highlight the continuous feedback and interdependence between phases, ensuring adaptability and improvement across the system. The figure was prepared using Microsoft PowerPoint and Icons adapted from Flaticon (www.flaticon.com) (accessed on 1 September 2025).

A preparedness framework encompasses key components such as the value chain stages of outbreak detection, confirmation, and response, along with cross-cutting enablers like financing, coordination, and human capacity (Figure 2). It emphasizes the importance of research, manufacturing, and in-country implementation for diagnostic readiness based on Perkins et al. [146]. Furthermore, it underscores the need to address both known and unknown pathogens with tailored activities and effective outbreak response measures. As a result, various industries and academic institutions are working on the development of quick, user-friendly, and POC diagnostic kits to ensure sensitive and specific viral detection. These diagnostic approaches can be categorized into two main groups: nucleic acid-based diagnosis and serology-based diagnosis. While diagnostics are a fundamental component of outbreak preparedness, this review offers a strategic overview rather than detailed technical specifications of individual diagnostic methodologies. We present a broad summary of key approaches—such as nucleic acid-based tests, serological assays, and emerging technologies—to illustrate their roles in early detection, surveillance, and coordinated response efforts. More detailed discussions of specific diagnostic methods are reviewed in [147,148].

Diagnostic development can be characterized by Technology Readiness Levels (TRLs), a scale (1–9) for maturity from basic research to fully proven products (U.S. Department of Health and Human Services, n.d.). In pandemic preparedness, high TRL (≈9) means widely available, licensed tools; whereas lower TRL indicates early or prototype stages. For example, the COVID-19 experience shows SARS-CoV-2 diagnostics (PCR and rapid tests) reached TRL ≈ 9, while many other pathogens lack such readiness. Table 4 summarizes representative viral categories, demonstrating that mature diagnostic platforms (PCR, antigen tests) score high, whereas novel or emerging diagnostic tools score lower.

### 4.1. Nucleic Acid-Based Diagnosis

The gold standard method for detecting viral nucleic acid is (RT)-qPCR. However, its technical accuracy has not yet resulted in effective SARS-CoV-2 containment during the early pandemic phase, highlighting the complexity of outbreak control beyond diagnostic capability [150]. In February 2020, the FDA expanded the EUA to enable qualified laboratories to develop and deploy in-house SARS-CoV-2 diagnostic assays [151], primarily performed in hospital and reference laboratory settings [152]. While RT-qPCR demonstrates high sensitivity and specificity under optimal conditions, its performance can be compromised by several factors. Significant variations in viral RNA sequences can influence results from RT–qPCR utilizing different primer sets targeting various parts of the viral genome, potentially leading to false negatives due to viral evolution. Also, PCR sensitivity might be reduced in the case of multiplex detection.

Another format is droplet digital PCR (ddPCR), which offers the absolute quantification of the target gene in samples without a standard curve. The technique relies on partitioning samples into thousands of individual droplets, enabling more precise quantification than conventional methods. Moreover, research indicates that ddPCR demonstrates superior sensitivity and specificity in identifying viruses within samples with low RNA abundance [153]. A comparative study examining the sensitivity of RT-qPCR versus ddPCR of ORF1ab and N genes of SARS-CoV-2 revealed positive ddPCR signals in 26 samples that were negative via RT-qPCR [154]. Similarly, Yu et al. [155] studied the ORF1ab and N genes of SARS-CoV-2 in different samples. They found that ddPCR was better than RT-qPCR at identifying the virus in samples with low viral loads. Nonetheless, it is important to note that ddPCR entails expensive instrumentation and longer turnaround times compared to RT-qPCR for obtaining results.

Isothermal amplification of nucleic acid is an alternative to nucleic acid amplification based on thermal cycling. The isothermal process makes the amplification method in POC diagnostic equipment possible and helps to develop virus RNA detection methods in resource-limited areas. Isothermal amplification reactions can be classified into three types depending on the reaction kinetics, including linear, exponential and cascade amplification. Among these isothermal amplification reactions, exponential amplification has a higher efficiency and detection sensitivity. Many exponential amplification methods have been proposed, such as loop-mediated isothermal amplification (LAMP), strand displacement amplification (SDA), and rolling circle amplification (RCA). Taking the hand-foot-and-mouth disease (HFMD) virus as an example, we systematically summarized the current status of isothermal nucleic acid amplification techniques for HFMD and discussed the advantages and drawbacks of various isothermal amplification processes [156].

The LAMP technique has attracted considerable attention because it enables rapid, sensitive, and specific detection under constant temperature, thereby eliminating the need for complex thermal cycling equipment. In this context, Abbott Diagnostics has introduced a POC device utilizing RT-LAMP for the detection of SARS-CoV-2 RNA in respiratory swabs [157]. LAMP method offers rapid results, with a turnaround time of just 13 min. However, its limitation to processing only one sample per run hinders the scalability of the technique. Numerous studies have highlighted the superior or comparable performance of LAMP compared to RT-qPCR for COVID-19 diagnosis. Coupled with its simplicity, speed, and POC capabilities, RT-LAMP stands out as an outstanding alternative to RT-qPCR [158]. Notably, smartphone-based LAMP assay demonstrates comparable sensitivity and quantitative accuracy to RT-qPCR for both SARS-CoV-2 and influenza virus detection [159]. Apart from smartphones, different isothermal approaches can be combined with the amplification reaction in a multichannel readout, such as microfluidic devices.

Due to the high-fidelity recognition of Clustered Regularly Interspaced Short Palindromic Repeats (CRISPR)/Cas technology, which offers single-base resolution and powerful, flexible signal transduction through efficient trans-cleavage, such as CRISPR/Cas13a for virus RNA recognition, it has become a promising method for virus detection. However, the sensitivity of CRISPR/Cas may be insufficient for viral detection in environmental samples due to low viral concentrations. Therefore, some studies have attempted to combine CRISPR/Cas with isothermal amplification, utilizing the specificity of CRISPR/Cas to solve the false positive problem of isothermal amplification and using the high sensitivity of isothermal amplification to improve the LOD of CRISPR/Cas. For example, we developed a portable paper device based on CRISPR/Cas12a and RT-LAMP with 97.7% sensitivity and 82% semiquantitative accuracy for SARS-CoV-2 detection in wastewater, highlighting a promising point-of-use approach for WBS [160].

### 4.2. Immunological Assays

Enzyme-linked immunosorbent assay (ELISA) is the most commonly used technique for serological testing across various diseases. However, cross-reactivity among antibodies elicited by closely related pathogens can complicate interpretation, and ELISA results should be considered preliminary rather than confirmatory. As demonstrated by Maeki et al. [161], cross-reactivity among flavivirus antibodies can produce false-positive ELISA results, and the use of paired serum neutralization tests is essential for accurate interpretation [161], illustrating the importance of confirmatory testing beyond standard ELISA [162].

ELISA allows for the identification of multiple antigenic proteins or various pathogens (referred to as multiplexing). Automation of this method enables high-throughput analysis and the capability for POC detection [163]. High-throughput automated ELISA demonstrates superior performance for large-scale diagnostic applications. Examples of high-throughput automated ELISA include Luminex X-Map and Simoa technologies that can be adapted for the multiplex detection of viral antigens or antibodies. Luminex X-Map technology represents a revolutionary advancement in multiplex immunoassay, utilizing spectrally distinct fluorescent microspheres to enable the simultaneous detection of multiple targets, including both proteins and nucleic acids [164,165,166]. Single Molecule Array (Simoa) technology provides unprecedented sensitivity enhancement, improving detection capabilities over 1200-fold compared to conventional ELISA. Simoa platforms achieve single-molecule sensitivity in the attomolar range (10^−16^ M) compared to conventional immunoassay detection limits in the femtomolar range (10^−13^ M). Automated Simoa instruments demonstrate throughput of 66 samples per hour with coefficients of variation below 10% [167].

The lateral Flow Assay (LFA) is a rapid diagnostic technique for qualitative serological analysis. Results can typically be obtained within 10–30 min, making it feasible for testing in POC setting. The rapid analysis, cost-effectiveness, and minimal need for specialized personnel make LFA well-suited for the extensive sample screening demanded during a pandemic [168,169]. It is also a self-testing antigen (Ag) test that played a significant role in the containment of COVID-19. Systematic reviews of COVID-19 lateral flow devices reveal sensitivity ranging from 64% to 76% across different commercial assays, while specificity consistently remains high, with 4 out of 5 LFAs achieving 100% specificity [170].

Generally, serological tests provide swift, cost-efficient, and on-site detection capabilities, making them ideal for high-throughput testing during outbreaks. As a result, (qRT)-PCR is often used as a confirmatory test, particularly when serological tests yield negative results in symptomatic cases. Despite these limitations, serological tests provide valuable insights into population-level immunity status and remain essential components of comprehensive diagnostic preparedness strategies.

Beyond manual lateral flow rapid antigen tests, analyzer-based immunochromatographic platforms offer automated signal interpretation and improved standardization. The Sofia 2 SARS Antigen Fluorescent Immunoassay (FIA) represents the primary widely deployed analyzer system that employs optical fluorescence detection with algorithmic interpretation of signal intensity, thereby reducing operator subjectivity and eliminating visual misreading that can occur with colorimetric lateral flow tests [171,172]. While Abbott Binax NOW is a rapid point-of-care immunochromatographic antigen test, it is a manual lateral flow assay that requires visual interpretation rather than an automated instrument-based system.

In terms of performance, analyzer-based systems like Sofia 2 FIA demonstrate performance substantially dependent on sample viral load and symptom status. Overall sensitivity ranges from 60.5% to 94.2% across populations, with sensitivity reaching 87–99.1% in samples with high viral loads (RT-PCR Ct ≤ 25) but declining to 28.7% or lower for low viral load samples (Ct > 30) [171,173]. Asymptomatic individuals demonstrate particularly low sensitivity: Sofia 2 achieved 41.2% sensitivity in asymptomatic populations despite specificity >98% [174]. When compared directly to high-quality manual lateral flow tests, analyzer-based fluorescence detection shows modest superiority primarily at high viral loads; both formats achieve >90% sensitivity in high viral load samples and <30% sensitivity in low viral load samples [175]. Critically, antigen test sensitivity declines over the course of infection: Abbott Binax NOW demonstrated 96.3% sensitivity in initial testing but declined to 48.4% in repeat testing 7–14 days later, reflecting waning viral shedding during recovery [176].

Regarding deployment, analyzer-based systems have been deployed in healthcare facilities, occupational health settings, and high-volume screening venues during the COVID-19 pandemic. However, these systems require more substantial infrastructure compared to manual rapid antigen tests, including electrical power, instrument calibration, maintenance contracts, and initial capital investment ($3000–$10,000), substantially limiting deployment in resource-limited settings where rapid diagnostic capacity is most needed [172,177].

Looking ahead, emerging technologies for antigen detection include electrochemical impedance spectroscopy-based biosensors, surface plasmon resonance sensors, and other label-free detection platforms that measure changes in electrical properties or optical signals when an antigen binds to immobilized antibodies. These platforms offer potential advantages of high sensitivity (detection limits from 0.99 picogram/mL to 200 nanogram/mL depending on platform design, with select systems achieving sensitivity approaching or exceeding conventional ELISA), rapid analysis (typically 10–30 min), and minimal sample preparation [178,179]. While certain systems have achieved clinical validation in laboratory settings, demonstrating 99% sensitivity and 100% specificity, most diagnostic platforms remain in development or early clinical validation phases and have not yet transitioned to routine point-of-care implementation or widespread clinical deployment.

The WHO’s prioritized research agenda for diagnostic technologies, including the Blueprint Pathogen X Research Agenda (2022) and the July 2024 updated priority pathogen list, targets ‘Prototype Pathogens’ to accelerate the development of point-of-care diagnostics and therapeutics. This approach reflects a strategic recognition that timely detection of unknown or emerging pathogens is essential for global health security. By emphasizing rapid, accessible diagnostics, particularly in resource-limited settings where zoonotic spillovers are most likely, the agenda directly informs capacity-building priorities, resource allocation, and the design of surveillance systems to mitigate outbreak risk before widespread transmission occurs.

### 4.3. Impact of Poor Diagnostic Capacity

Limited diagnostic capabilities in healthcare systems can seriously impede outbreak control efforts. This was evident during the 2016–2017 yellow fever (YFV) outbreak in Central Africa [180]. Despite the endemic nature of YFV across Africa and the availability of an effective vaccine for nearly eight decades, the preparedness for the outbreaks in Angola and Nigeria during this period was inadequate [181]. Although detecting YFV through ELISA was technically possible at the national level, the shortage of crucial reagents prevented labs from testing most suspected cases. The minimal case reproduction numbers observed during the Ebola outbreak indicate that even slight enhancements in interrupting transmission can significantly impact disease control. Additionally, during the 2013–2016 Ebola epidemic in West Africa, it took three months from the emergence of the index case to the identification of the causative agent. A post-epidemic modeling study found that if 60% of Ebola patients were diagnosed within a day instead of five days, the percentage of people infected in the population (attack rate) could have dropped from 80% to nearly zero [182]. These findings underscore that even modest improvements in diagnostic speed can dramatically enhance transmission interruption and disease control outcomes.

Influenza research has led to numerous laboratory tests for identifying influenza and its subtypes, allowing for rapid development of testing for novel strains, particularly for pre-pandemic strains. Human cases of pre-pandemic strains are quickly identified and monitored to contain potential pandemic outbreaks. Despite this success, the delayed development and distribution of diagnostic testing kits for SARS-CoV-2 led to delayed case identification and contact tracing, causing widespread viral transmission. Interestingly, SARS-CoV-2 detection in the USA occurred two months after its initial identification in China, resulting in delays in implementing RT-qPCR tests and facilitating viral transmission. Hence, there is a need for unbiased detection methods that do not rely on viral sequence data to diagnose infections, such as metagenomics analysis [183]. This approach could potentially identify unknown pathogens during the critical early phases of outbreaks when conventional sequence-based diagnostics are unavailable. However, implementation of this technology requires significant infrastructure investment and technical expertise, which may be lacking in resource-limited settings where many outbreaks originate.

Together, the absence of accessible and definitive diagnostic tools has been identified as a critical gap in global health security infrastructure. The lack of accessible and definitive diagnostic tools is especially critical for outbreaks that emerge in rural areas, such as Lassa fever, or affect mobile populations, like MERS-CoV, where limited infrastructure and rapid population movement exacerbate transmission risks. Common critical obstacles to diagnostic preparedness and potential remedies are presented in Table 5, modified from Kelly-Cirino et al. [184], summarizing key challenges in research, development, logistics, and healthcare systems along with proposed solutions.

## 5. Prevention and Control Measures

### 5.1. Vaccination

Vaccines fall into two main categories: conventional and next-generation platforms. Conventional vaccines utilize traditional approaches, including live-attenuated, inactivated, or subunit formulation derived from weakened or killed pathogens. While these platforms are effective for numerous diseases, they exhibit limitations when confronting complex pathogens and require extended development timelines. Next-generation vaccines represent a paradigm shift in vaccine development, employing advanced biotechnological approaches including genetic engineering, viral vectors, and nucleic acid-based platforms. These approaches offer a faster, potentially more effective and adaptable solution to emerging pathogens, exemplified by rapid COVID-19 vaccine development [185,186].

The rapid development of COVID-19 vaccines can be attributed to two decades of focused research on coronaviruses following the 2002 SARS-CoV outbreak in Asia and the 2012 MERS-CoV emergence in the Middle East. Reflecting on the SARS-CoV-1 outbreak in 2002, it is evident that despite its relatively low number of deaths and infections, its high mortality rate and ease of transmission resulted in substantial global disruption. The epidemic concluded, coinciding with the commencement of vaccine development efforts. Subsequently, with the closure of wet markets and the cessation of human-to-human transmission from civets, the disease has not resurfaced. Consequently, research on SARS-CoV-1 vaccines was discontinued, and funding for such efforts was reduced.

**Figure 3:**
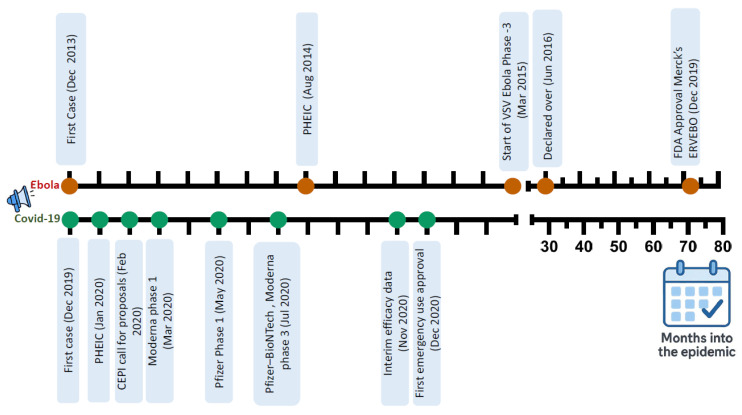
Timeline comparison of Ebola (2014–2016) and COVID-19 (2019–2020) outbreaks showing key milestones in case detection, public health emergency declaration, vaccine clinical trials, and regulatory approvals. The Ebola vaccine (ERVEBO) required nearly six years from the outbreak onset to regulatory approval in 2019. In contrast, COVID-19 vaccines were granted emergency use authorization within one year of the pandemic’s emergence, highlighting a paradigm shift in vaccine development enabled by advanced biotechnology, rapid clinical trial designs, and global collaboration.

While the 2014 Ebola epidemic lasted over two years and caused over 11,000 deaths, it allowed sufficient time for the development and clinical evaluation of various vaccines (Figure 3). By the epidemic’s end, one of these vaccines, the rVSV-ZEBOV vaccine, demonstrated high efficacy in ring vaccination trials. In contrast, the COVID-19 pandemic presented an unprecedented timeline challenge: the entire process, from identifying the virus and genome sequencing to analyzing early data on vaccine efficacy, was completed remarkably quickly, within a year [187].

#### 5.1.1. Vaccine Platform Technologies

The rapid deployment of vaccines directly impacts outbreak control. However, the traditional vaccine development process is unsuitable for rapid responses during explosive pandemics. Fortunately, new vaccine platform technologies have the potential to shorten this cycle, enabling faster development, testing, and production of multiple vaccines [188].

Notably, two COVID-19 vaccines—Pfizer–BioNTech and Moderna utilize mRNA technology, demonstrating safety and high efficacy [185,186]. These vaccines have been authorized for emergency use only by the FDA [189] and conditional marketing authorization from the European Medicines Agency (EMA) [190,191,192]. While mRNA vaccines are promising for other emerging infectious diseases, it remains premature to declare them a universally applicable approach. The COVID-19 mRNA vaccines serve as a valuable proof of concept, but further research and real-world effectiveness assessments are necessary.

Although numerous DNA vaccines are licensed for veterinary use and have demonstrated safety and effectiveness in human clinical trials, none have been officially approved for human use [193]. Recombinant proteins, encompassing diverse designs for specific pathogens, such as subunit vaccines or virus-like particles (VLPs), are often combined with adjuvants to enhance immunogenicity but generally require longer development timelines. VLP vaccines for hepatitis B and human papillomavirus demonstrate safety and efficacy profiles, while offering scalable manufacturing through technology that can be easily transferred [194].

On the other hand, whole inactivated virus vaccines (such as CoronaVac for SARS-CoV-2 and PV vaccine) or attenuated vaccines (including PV and yellow fever vaccines) require pathogen-specific development approaches. However, production of certain inactivated vaccines, including SARS-CoV-2 and some influenza strains, may require biosafety level 3, limiting global production scalability [195].

Other vaccine platforms use recombinant vectors, which are either non-replicating vectors (like adenovirus 5 (Ad5), Ad26, and ChAdOx) or live attenuated vectors (like the measles virus-based vectors). These vector-based vaccines can be modified by adding pathogen-specific genetic inserts while maintaining the vector backbone. Non-replicating adenoviral vectors are promising vaccine platforms due to higher yield, cGMP-friendly manufacturing processes, safety, enhanced efficacy, and manageable shipping and storage procedures [196].

To achieve optimal protection against diverse viral threats, comprehensive preparedness requires a diverse portfolio of vaccines based on different platform technologies. These platforms have the potential to address vaccine manufacturing challenges, including speed, safety, and efficacy, as well as ongoing monitoring of their effectiveness across different pathogen families [196,197]. The vaccine readiness landscape, summarized in Table 4, illustrates this gap: while established platforms for well-known pathogens (influenza, measles, PV) have reached TRL ≈ 9, many emerging viral families still lack licensed vaccines or remain in early development phases.

Experience with these platforms in recent outbreaks—most notably the 2009 H1N1 pandemic and COVID-19—demonstrated how prior platform development and regulatory preparedness directly influence the speed and effectiveness of vaccine deployment. The COVID-19 pandemic and other recent outbreaks underscore the importance of proactive, multi-sector collaboration, especially when there is clear alignment of objectives, leadership, and resources toward public health goals. Effective pandemic preparedness requires sustained partnerships between regulators, industry, academia and international organizations to create clear, responsive, and adaptive guideline systems capable of rapid deployment during emergencies.

In 2009, a novel H1N1 influenza strain emerged, causing concern about its potential severity. Unlike recent outbreaks of COVID-19, Zika, and Ebola, the existing seasonal influenza vaccine infrastructure was available, though single-dose effectiveness against H1N1 was uncertain. Drawing upon experiences with avian influenza threats like H5N1, many experts initially expected to require two doses and novel adjuvants for effective immunization. To expedite the 2009 H1N1 vaccine development, the FDA implemented a key regulatory strategy based on prior guidance. The FDA clarified that vaccines manufactured using existing methods and facilities for seasonal influenza, with strain-specific adjustments for the new H1N1 strain, would be considered “strain-change modifications” of existing licensed vaccines rather than entirely new biological products [198]. This eliminated the need for large clinical trials, significantly accelerating and simplifying the development process.

The 2009 pandemic demonstrated both the potential and limitations of regulatory approaches. Despite streamlined regulatory processes, vaccine development and production timelines remained insufficient to influence the first wave of the pandemic or meet global demand [199]. This inefficiency underscores the limitations of existing regulatory frameworks and manufacturing capacity in responding to emergent threats. Lessons learned emphasized the need for advanced regulatory science, long-term platform investments, and flexible manufacturing capacity to address diverse threats. These insights directly informed the development of next-generation platforms, such as mRNA vaccines, which demonstrated rapid efficacy in the initial COVID-19 vaccine response, validating the importance of adaptable technologies in pandemic preparedness.

#### 5.1.2. Potential Use of Preparedness Vaccines

Preparedness vaccines, also called “prototype”, “broad-spectrum” or “next-pandemic” vaccines, are designed to protect against multiple viral strains within a particular family or across different viral families. These vaccines are particularly relevant for addressing Disease X and Pathogen X, providing a framework for rapid response against unknown or emerging pathogens. These vaccines are typically developed based on conserved regions of viral proteins that are shared across strains, enabling cross-protective immunity [200]. Challenges include predicting emerging strains and securing funding and infrastructure; however, vaccine technology advancements and increased global collaboration have enabled feasible development.

Taking the influenza virus as a model, current seasonal influenza vaccines are strain-specific, and their effectiveness varies yearly because the dominant strain continuously evolves through antigenic drift [201]. High-performance seasonal vaccines offer strong immunity against matched strains but lose potency when viruses undergo significant antigenic drift or when novel pandemic strains emerge. The influenza vaccine preparedness spectrum encompasses several distinct categories:

Supraseasonal vaccines consistently protect against antigenically drifted (mismatched) seasonal viruses over multiple years, though their efficacy against pandemic strains may remain limited [200]. Prepandemic stockpile vaccines are primarily effective when pandemic viruses closely match the stockpiled strain.

Pandemic preparedness vaccines are designed to provide broad cross-protective immunity against both seasonal and pandemic viruses. While their effectiveness may be lower than strain-matched vaccines, these vaccines enable rapid deployment during outbreaks, offering a partially effective intervention before a tailored vaccine can be produced.

Pandemic response vaccines provide substantial protection against emerging pandemic viruses by leveraging adaptable platform technologies that enable rapid development, based on the genetic sequence of new pathogens. Universal influenza vaccines designed to provide comprehensive and enduring protection against both circulating seasonal strains and emerging pandemic viruses, overcoming the limitations of current seasonal vaccination [200].

Multiplex Vaccines:

Multiplex vaccines are designed to protect against multiple viruses simultaneously. These vaccines can be especially useful in regions where multiple viral pathogens co-circulate or in anticipation of potential emerging threats. Researchers developed a new approach using a chimeric DNA construct combining parts of influenza and MERS-CoV viruses. This resulted in a novel vaccine strain potentially offering protection against both H1N1pdm09 and MERS-CoV [202]. The effectiveness of lipid nanoparticle mRNA (LNP-mRNA) vaccines against COVID-19 prompted speculation about the potential for multiplexed vaccination targeting various coronavirus species. Previous research in other virus families, such as influenza, herpes simplex virus (HSV), and cytomegalovirus (CMV), provided initial evidence of the feasibility of employing multiple mRNA vaccine constructs in combination [203,204,205]. Early research explored the use of mRNA vaccines that combine multiple antigens of CMV, influenza, and HSV-2. Scientists are also interested in developing multi-species coronavirus vaccines.

Recent studies have explored the use of chimeric mRNA or the combination of protein antigens to develop vaccines that target two or more coronaviruses [206,207]. Martínez et al. [207] focused on generating chimeric vaccine constructs for SARS-CoV, SARS-CoV-2, and other non-pathogenic species (e.g., HKU3-1 and RsSHC014), excluding MERS-CoV. Nevertheless, chimeric spikes may not encompass all full-length spikes, potentially missing critical antigenic regions (e.g., S1 or S2) for certain species—notably, the two mRNA vaccines approved for use utilized full-length spike proteins [185,186]. Peng et al. [208] produced LNP-mRNA constructs to encode the HexaPro engineered full-length spikes derived from SARS-CoV-2, SARS-CoV, and MERS-CoV. Moreover, exploring the optimal vaccination schedule for multiplexed vaccination is crucial, including determining the effectiveness of administering all mRNAs simultaneously versus spacing out the administration of different mRNA vaccine shots.

Another example is the study by Arevalo and colleagues, who used an unconventional approach, encoding 20 different influenza HA antigens that span both A subtypes and B lineages, representing the genetic diversity of the virus. When mice were immunized with these 20 HA antigens, they produced neutralizing antibodies against desirable group 1 and group 2 HA stalk/stem epitopes. Interestingly, T cell depletion did not impact vaccine efficacy, emphasizing the critical role of antibodies in protection. The vaccine protected mice and ferrets from severe disease even when challenged with influenza strains not covered by the vaccine [209].

While these approaches are promising, challenges include predicting which pathogens will emerge, striking a balance between breadth and efficacy, and ensuring sufficient production capacity. Multiplexed vaccines, such as chimeric mRNA constructs targeting multiple coronaviruses or influenza strains, demonstrate feasibility but face technological, regulatory, and logistical hurdles. Even universal vaccines, while offering long-term protection against multiple variants, may not fully prevent infection by unexpected strains and require careful integration into global preparedness strategies. Despite the promise of prototype and broad-spectrum vaccines, significant limitations remain. Prioritizing which pathogen families to target requires careful risk assessment based on epidemic potential, transmissibility, and population vulnerability, as exemplified by the WHO Blueprint for priority diseases. Cross-protective immunity may be incomplete, and some pathogens can evolve unpredictably, thereby challenging the breadth and efficacy of the vaccine. Limited preclinical and clinical data for emerging virus families can further delay readiness, underscoring the need for continued surveillance, research, and adaptive development strategies.

Universal Vaccines:

Universal vaccines aim to provide long-lasting protection against multiple strains or types of viruses within a particular family. These vaccines target conserved regions of viral proteins and can potentially offer cross-protection against a range of viral variants. For the context of this analysis, a universal influenza vaccine is defined as one capable of eliciting protective immunity against all influenza A and B viruses. Therefore, the study by Arevalo et al. [209] could be a promising step toward a universal influenza vaccine. The effectiveness of a universal vaccine would remain consistent over multiple years, ideally extending beyond three years, with a minimum 75% reduction in medically attended lower respiratory tract disease or hospitalization. This implies that the vaccine would not require annual adjustment and would sustain immunity against both seasonal and pandemic viruses that undergo genetic drift. This differentiation from a supraseasonal vaccine is characterized by its broader coverage of pandemic subtypes and its ability to confer protective immunity lasting more than one year with a single dose. Consequently, a universal influenza vaccine would exhibit superior efficacy compared to presently available vaccines when matched with prevalent strains, suggesting heightened potency or a more favorable anatomical site for inducing protective antibody and T-cell responses [210].

The Computationally Optimized Broadly Reactive Antigen (COBRA) approach has been employed to design HA and neuraminidase antigens that protect a broad spectrum of strains within each subtype [211]. This approach shows promise as a candidate for a universal influenza vaccine, capable of triggering protective immune responses against both seasonal and pre-pandemic strains across multiple seasons. The COBRA methodology effectively tracks antigenic evolution while preserving conserved epitopes to safeguard against re-emerging viruses, presently circulating strains, and potential future variants [212]. Moreover, the COBRA immunogen can be integrated into various delivery platforms, including mRNA and viral vectors, enabling flexible deployment.

#### 5.1.3. Vaccine Safety Monitoring and Adverse Effects

WHO and other regulatory authorities emphasize that COVID-19 vaccines have excellent safety profiles: serious adverse reactions are very rare, and vaccination remains far safer than infection [213]. In practice, most vaccine recipients report only transient local and systemic reactions—for example, fever, myalgia, headache or sore arm—that typically resolve within 1–2 days [214]. Severe allergic reactions (anaphylaxis) are rare, occurring in only a few reported cases per million doses administered [214].

Even rarer still are the specialty-associated events that have dominated recent safety surveillance, which include risks from different vaccine platforms. For example, mRNA vaccines can very occasionally trigger myocarditis or pericarditis, mainly in young males, at roughly 1–5 cases per 100,000 doses [213], while adenovirus-vector vaccines have been linked to vaccine-induced thrombotic thrombocytopenia (VITT/TTS) at rates of around 7–8 per million first doses [215] and to Guillain–Barré syndrome at approximately 5–7 per million [216].

Large-scale cohort studies of updated booster vaccines have found no new safety signals. For instance, a Danish analysis revealed no significant increase in any of the 29 serious events (incidence rate ratio for myocarditis ≈ 1.12, 95% CI 0.41–3.10) in the month following vaccination [217]. Taken together, these data imply that most vaccine side effects are mild and predictable, while severe events remain vanishingly rare. For outbreak preparedness, this means that vaccination programs should incorporate robust surveillance and communication about both expected reactogenicity and rare risks so that health systems can manage these events and maintain public confidence in mass immunization.

#### 5.1.4. Key Considerations in Vaccine Development and Regulatory Evaluation

In addition to proving safety and efficacy, successful vaccine development necessitates thorough evaluation of manufacturing scalability, logistical feasibility, global accessibility, regulatory frameworks, and cost-effectiveness to achieve mass immunization. Critical prerequisites include logistical feasibility (transport, storage, and administration across diverse settings) and technology transfer, enabling global production [218].

Many next-generation platforms, particularly lipid nanoparticle mRNA vaccines, require ultra-cold storage (−70 °C for Pfizer/BioNTech), creating substantial barriers for distribution in LMICs and remote areas. Viral vector vaccines have less stringent cold-chain needs (2–8 °C), but maintaining consistent temperature control remains challenging in resource-limited settings. These constraints can delay deployment during outbreaks and limit the speed of achieving population-level immunity.

Global vaccine production remains concentrated in a limited number of countries. High-income nations often secure the majority of early doses, while LMICs rely on donations or delayed deliveries. The COVID-19 pandemic exemplified how these disparities prolong outbreaks, contribute to the emergence of variants, and undermine the global public health response. Enhancing local manufacturing capacity, technology transfer, and sustainable funding for vaccine production are essential strategies to reduce inequity.

Large-scale production is a critical obstacle in pandemic preparedness, requiring sophisticated equipment, specialized facilities, and highly trained personnel [219]. Only a limited number of countries possess independent manufacturing capabilities, restricting global vaccine availability [220]. Cost-effectiveness is essential to ensure broad access, influenced by technological platforms, development and manufacturing costs, public funding, licensing strategies, and company profit objectives [218,221]. Additionally, regulatory frameworks play a pivotal role in enabling rapid vaccine deployment during emergencies. Experience from the 2009 H1N1 pandemic and COVID-19 illustrates that prior platform knowledge can accelerate approval processes by allowing vaccines to be considered “strain-change modifications” rather than new products. EUAs facilitate early use based on preliminary evidence of efficacy and safety, but must be balanced with rigorous post-market surveillance [222]. Regulators must also coordinate internationally to ensure equitable access while maintaining high standards of safety, efficacy, and quality. Clear, adaptive guidelines, supported by collaboration between regulatory agencies, industry, and international organizations, can mitigate delays and prevent bottlenecks during pandemic responses.

Beyond immediate safety and efficacy, vaccines also contribute to reducing long-term health consequences of viral infections, which should be considered in preparedness planning. Viral outbreaks can leave persistent health impacts beyond the acute phase. In COVID-19 survivors, post-COVID syndrome (“long COVID”) is characterized by fatigue, breathlessness, neurocognitive deficits, and multi-organ impairment lasting months or longer [223]. COVID-19 vaccination before infection is associated with protective effects against long COVID, with meta-analysis evidence showing that two-dose vaccination reduces the risk of long COVID compared to no vaccination (odds ratio, 0.64; 95% confidence interval, 0.45–0.92), and also reduces the risk of specific long COVID symptoms, including persistent fatigue and pulmonary complications [224]. Vaccination administered after SARS-CoV-2 infection shows that among individuals with ongoing long COVID symptoms, most do not experience symptomatic changes following vaccination, although approximately 20% report symptomatic improvement [225]. Incorporating long-term sequelae in epidemiological frameworks and health economic models enables better assessment of the full benefits of prevention measures and informs public health planning. Effective epidemic management requires a robust infrastructure for information exchange, international collaboration, and knowledge sharing [226].

### 5.2. Antiviral Therapeutics

Beyond vaccination, comprehensive preparedness strategies should include antiviral therapeutics. While vaccines play a crucial role in preventing infections, antivirals offer unique and essential benefits in different stages of outbreak management. Viral disease progression occurs in two phases: an early viral phase, which can be managed by antiviral medications, and a later hyperinflammatory phase, which requires host-targeted immunomodulators.

**Figure 4:**
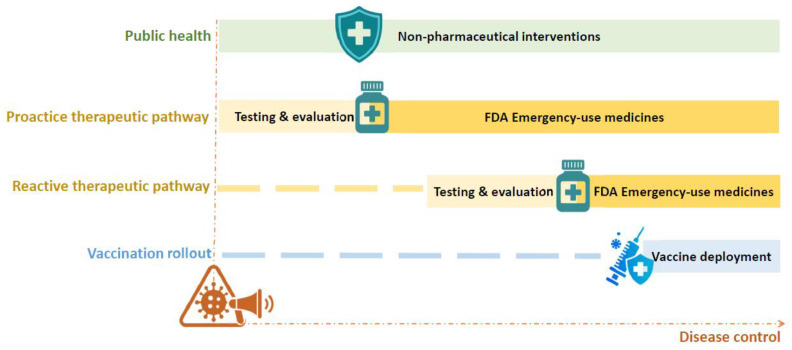
Timeline of response strategies during a novel viral outbreak, from identification to disease control. The initial response prioritizes public health interventions, such as social distancing, masking, and contact tracing, which act as the first line of defense to slow transmission and buy time for therapeutic development. The proactive therapeutic pathway represents efforts to test and evaluate potential antiviral drugs before or early in an outbreak, allowing faster deployment of FDA emergency-use medicines and reducing morbidity and mortality. The reactive therapeutic pathway illustrates the typical sequence where therapeutic evaluation and drug deployment occur after the outbreak has escalated, highlighting delays in controlling disease progression. Vaccination rollout, shown as a later-stage intervention, ultimately contributes to durable population immunity. The figure was prepared using Microsoft PowerPoint with icons adapted from Flaticon (www.flaticon.com) (accessed on 1 September 2025).

The epidemiological data guide targeted therapeutic interventions in areas where the risk is most significant, enabling more effective resource allocation and clinical outcomes. However, the development of new therapeutics and vaccines needs time, as shown in Figure 4. To better prepare for future outbreaks, researchers should aim to develop at least one therapeutic candidate for each virus family, with initial safety testing (Phase 1) completed for rapid deployment. Although challenging, this task is crucial for public health preparedness.

Identifying viruses with the highest potential for outbreaks is critical for therapeutic development. As emphasized earlier, continuous viral surveillance is essential. This includes monitoring circulating strains to explore changes in their sequence and spread, ultimately informing our preparedness. This epidemiological information set the stage for the research community to construct a portfolio of versatile therapeutics to combat emerging viral threats. Two main categories exist for this development: repurposing existing drugs or developing entirely new ones.

#### 5.2.1. Drug Repurposing Attempts

This approach prioritizes FDA-approved drugs, offering the fastest path to approval for new uses during an outbreak (Table 6).

While the pandemic accelerated the development of antiviral treatments, many promising candidates failed in rigorous clinical testing, providing vital lessons for future preparedness. Hydroxychloroquine (HCQ) emerged as a leading candidate in early 2020, receiving FDA EUA on 28 March 2020, based on in vitro studies against SARS-CoV-2. However, subsequent large randomized controlled trials, including the WHO Solidarity trial and the DisCoVeRy trial, found that HCQ provided no mortality benefit and actually increased the risk of gastrointestinal adverse effects, cardiac toxicity, and cognitive dysfunction in hospitalized patients [227,228,229]. As a result, the FDA revoked HCQ EUA on 15 June 2020, after analyzing accumulated clinical evidence.

Remdesivir, an adenosine nucleoside analogue originally developed for Ebola virus, initially generated substantial hope as a direct-acting antiviral. While remdesivir showed some benefit in reducing time to recovery in early, less severe COVID-19 cases, major randomized trials, including the DisCoVeRy trial, demonstrated limited or no mortality benefit in hospitalized patients with severe disease [230]. The WHO Solidarity trial reported that remdesivir had ‘little or no effect’ on hospitalized patients, showing no significant reduction in mortality, initiation of ventilation, or duration of hospital stay [229,231]. These results highlighted that the timing of antiviral administration is critical; antivirals appear to work only during the early viral replication phase before inflammatory complications dominate the disease course.

**Table 6:** Examples of repurposed drugs for viral infection.

Drug Name	Original Indication	Repurposed Viral Use	Mechanism of Action	Current Status	Reference
**Remdesivir**	Ebola virus treatment	COVID-19, SARS-CoV-2	RNA-dependent RNA polymerase inhibitor	FDA-approved for COVID-19	[232]
**Favipiravir**	Influenza (Japan)	COVID-19	RNA polymerase inhibitor	EUA in multiple countries	[233]
**Nitazoxanide**	Antiparasitic (Cryptosporidiosis)	Influenza, COVID-19	HA maturation inhibition; immunomodulation	Phase III for influenza	[234]
**Lopinavir/Ritonavir**	HIV protease inhibition	COVID-19, MERS-CoV	Viral protease inhibitor	Discontinued for COVID-19	[235]
**Hydroxychloroquine**	Malaria, autoimmune diseases	COVID-19 (early pandemic)	Endosomal pH modulation; immunomodulation	Withdrawn due to inefficacy	[236]
**Umifenovir**	Influenza (Russia/China)	COVID-19	Viral entry/fusion inhibition	Investigational (Phase III)	[237]
**Molnupiravir**	Investigational for RNA viruses	COVID-19	RNA mutagenesis (error catastrophe)	FDA/EUA approved	[238]
**Atorvastatin**	Hypercholesterolemia	Influenza adjunct therapy	HMG-CoA reductase inhibition; immunomodulation	Phase II completed	[239]
**Baricitinib**	Rheumatoid arthritis	COVID-19 cytokine storm	JAK-STAT pathway inhibition	FDA-approved for COVID-19	[240]
**Camostat mesylate**	Pancreatitis (Japan)	COVID-19	TMPRSS2 protease inhibition	Phase III trials ongoing	[241]
**Dexamethasone**	Inflammatory conditions	COVID-19 ARDS	Broad-spectrum anti-inflammatory	Standard of care for severe COVID-19	[242]
**Ruxolitinib**	Myelofibrosis	COVID-19 cytokine storm	JAK1/JAK2 inhibition	Investigational	[243]
**Interferon-β combination**	Hepatitis C, malignancies	COVID-19 combination therapy	Broad antiviral cytokine induction	Limited use in combinations	[244]
**Zapnometinib**	Cancer	Influenza virus/COVI-19	MEK Inhibitor/immunomodulation	Preclinical/phase II	[245]

It is noteworthy that the interim findings from the WHO solidarity clinical trial revealed that the antiviral combination lopinavir/ritonavir, the HIV-1 protease inhibitor (Kaletra), showed no mortality benefit in COVID-19 hospitalized patients and was discontinued from the WHO Solidarity trial due to futility [229]. Moreover, the DisCoVeRy trial showed no improvement in clinical status at day 15 for lopinavir/ritonavir, lopinavir/ritonavir plus interferon β-1a, or HCQ compared to standard care. Additionally, participants allocated to lopinavir/ritonavir-containing arms experienced significantly higher rates of serious adverse events [246]. Collectively, these repurposing efforts revealed critical lessons for pandemic preparedness: (1) In vitro antiviral activity does not guarantee clinical benefit—rigorous human trials are essential before widespread use; (2) Well-designed, coordinated clinical trials are crucial for rapidly identifying ineffective therapies; (3) Drug efficacy depends on disease stage, with antivirals most effective early and immunomodulators in later, hyperinflammatory phases; (4) Side effects can be severe, emphasizing the need for thorough safety knowledge before deploying treatments in critically ill patients.

Despite current limitations, combination therapy strategies retain theoretical potential for enhanced efficacy by targeting different viral mechanisms and reducing resistance development [247,248]. Therefore, high-throughput screening of approved and pipeline drugs against diverse viruses coupled with pre-defined combination regimens should be conducted to identify more effective candidates for future outbreaks.

#### 5.2.2. Direct-Acting Therapeutics Development

In contrast to repurposed agents, newly designed direct-acting antivirals have shown substantial clinical benefit, particularly in the outpatient setting. For instance, the FDA authorized two leading oral COVID-19 antiviral drugs in late 2021, namely molnupiravir and Paxlovid, representing advances in outpatient treatment capabilities. Molnupiravir, a prodrug of the broad-spectrum nucleoside analog N4-hydroxycytidine [249], targets the viral RNA polymerase, inducing lethal viral mutagenesis [250,251].

Clinical trials demonstrated that molnupiravir reduced the risk of hospitalization or death by approximately 30% when administered within 5 days of symptom onset [238]. However, concerns remain about potential mutagenic effects and the possibility of promoting viral mutations that could enhance transmissibility [252]. Paxlovid comprises nirmatrelvir, a peptidomimetic inhibitor of the viral main protease, combined with ritonavir, a cytochrome P450 enzyme inhibitor that increases nirmatrelvir levels [253].

Clinical and real-world studies demonstrated that nirmatrelvir/ritonavir (Paxlovid^®^) has superior efficacy compared to molnupiravir, reducing hospitalization or death by 88% and enabling more rapid viral clearance [254,255,256].

RNA-based therapeutics are emerging as a powerful new class of drugs with immense potential. Their modular design and ease of development make them attractive alternatives to traditional drugs. RNA can exert therapeutic effects through various mechanisms. For example, CRISPR effectors and guide RNAs (gRNAs), enable targeted destruction of cellular RNAs or precise gene editing [257]. Messenger RNA (mRNA) delivers genetic instructions for protein production, acting as therapeutic proteins or vaccines [186,258].

#### 5.2.3. Neutralizing Monoclonal Antibody Efficacy and Variant Escape

Another potential approach for outbreak preparedness involves the use of monoclonal antibodies (mAbs) as prophylactics or treatments. To develop mAbs, researchers typically utilize the B cells of patients who have been infected to produce antibody libraries and then screen for highly neutralizing antibodies [259]. However, before the emergence of a novel virus, human-derived antibodies may not be available, necessitating reliance on animal models or computational design approaches.

mAbs demonstrate the potential of an immunotherapeutic approach with documented success in some viral hemorrhagic fevers, but they face significant challenges from viral evolution and antibody escape. The initial randomized clinical trial evaluating monoclonal antibodies for Ebola virus disease revealed that combining monoclonal antibodies could provide a safe and effective therapy for severe viral hemorrhagic fever, establishing a foundational proof-of-concept for this treatment [260]. However, the COVID-19 pandemic exposed critical limitations of single monoclonal antibodies or narrow-spectrum combinations.

During the COVID-19 pandemic, multiple mAbs received emergency authorization, including bamlanivimab, casirivimab/imdevimab (REGEN-COV), sotrovimab, and others, which were deployed both therapeutically and prophylactically [261,262,263]. While initially effective against the original SARS-CoV-2 strain and early variants (Alpha, Beta), these mAbs rapidly lost potency against emerging variants of concern, particularly Omicron and its sublineages, because viral evolution generated mutations in the spike protein’s receptor-binding domain and N-terminal domain [264,265,266]. Studies demonstrated that SARS-CoV-2 spike protein variants with mutations conferring resistance to mAbs and convalescent plasma could be readily selected in vitro and were already present at low frequencies in circulating virus populations during 2020 [267]. The mechanisms of antibody escape involve specific amino acid substitutions at residues critical to antibody binding. Single-residue changes can enable viral escape from mAbs without substantially compromising replication fitness, and different mutations can mediate escape from different antibodies targeting overlapping epitopes.

Moreover, these antibodies hold significant promise for treating severe cases of influenza virus infection, including those caused by zoonotic strains like H5N1 or H7N9 [268,269,270,271]. Given the diversity among viral strains and the unpredictable nature of future outbreaks, it is crucial to prioritize the development of therapeutics with broad activity by targeting conserved viral replication factors. Regardless of their mechanisms, all potential therapeutics should undergo comprehensive evaluation against a range of viral strains, encompassing both historical and contemporary variants. This testing strategy ensures therapeutic candidates maintain effectiveness against viral evolution and provides confidence in their pandemic response utility.

This “evolutionary arms race” between neutralizing antibodies and viral variants highlights key preparedness lessons: (1) mAbs targeting single viral sites are easily evaded, as seen with bamlanivimab and casirivimab/imdevimab losing efficacy. (2) Using antibody cocktails that target multiple conserved sites can reduce resistance. (3) Ongoing genomic surveillance is vital to detect escape variants quickly. (4) Developing broadly neutralizing antibodies against conserved viral regions should be a priority to limit future escape.

#### 5.2.4. Host-Targeted Therapeutics Development

Beyond direct-acting antiviral agents, host-directed therapeutics have emerged as critical components of severe viral infection management, exploiting the host’s own biology to limit viral replication or excessive inflammation in ways that direct-acting antivirals cannot.

Interferons (IFNs) represent the first-line immunomodulatory defense against viruses, with Type I interferon (IFN-α/β) activating interferon-stimulated genes (ISGs) with potent antiviral properties and documented efficacy against chronic hepatitis C virus (HCV) when combined with ribavirin, providing the first curative treatment for HCV [272]. However, the timing of interferon administration proved critical: early in infection, antiviral effects of interferons may provide protection, whereas late interferon treatment may enhance immunopathology and inflammatory lung injury, highlighting a fundamental timing-dependent paradox where the exact immune mechanism can be protective or harmful depending on disease stage.

Similarly, severe COVID-19 is characterized by excessive inflammatory cytokine production (cytokine storm), particularly elevated IL-6 levels correlating with severe disease and mortality [273], where tocilizumab, an IL-6 receptor antagonist monoclonal antibody originally developed for rheumatoid arthritis, demonstrated reduced mortality in hospitalized COVID-19 patients with hyperinflammation [274]. However, other clinical trials reported that tocilizumab administration did not lead to significant improvements in clinical outcomes or reductions in mortality rates [275,276]. Unlike antivirals, tocilizumab targets the inflammatory sequelae of infection rather than viral replication itself, allowing use during advanced disease stages where viral load is declining but inflammatory complications predominate, though other IL-6 pathway inhibitors and TNF-α antagonists showed variable efficacy, suggesting that cytokine pathway complexity limits single-target anti-cytokine approaches.

In addition, JAK inhibitors represent a downstream pharmacological approach to broadly inhibit cytokine signaling without targeting individual cytokine pathways, with baricitinib (a JAK inhibitor) combined with remdesivir shortening recovery time and reducing mortality in COVID-19 patients compared with remdesivir alone [277], and as orally bioavailable small molecules, they offer practical advantages over large-molecule antibody therapeutics that require intravenous administration. Corticosteroids (dexamethasone, methylprednisolone) reduce severe COVID-19 mortality by suppressing excessive inflammation, particularly in patients on oxygen or ventilatory support, with the RECOVERY and CoDEX trials definitively demonstrating that dexamethasone reduced 28-day mortality in hospitalized COVID-19 patients requiring oxygen support [242,278]. However, the RECOVERY trial found no benefit and even the possibility of harm among patients who did not require oxygen [242]. Early use of dexamethasone in COVID-19 patients not requiring oxygen or nasal cannula offered no mortality benefit and could cause harm due to immunosuppression during active viral replication; accordingly, guidelines contraindicated early corticosteroid use [279,280,281].

Future pandemic responses should use stage-specific treatment protocols: (a) early stage: start antivirals and interferon; (b) transition phase: shift from antivirals to anti-inflammatory therapies; (c) hyperinflammatory phase: use corticosteroids, IL-6 antagonists, and/or JAK inhibitors. Success depends on real-time clinical and immunological data, supported by investments in point-of-care biomarkers. Combining antivirals and immunomodulators increases safety monitoring needs, requiring pre-existing clinical trial infrastructure. Since many host-directed therapies are expensive or require injections, planning should prioritize affordable oral options and licensing for rapid production in low-income countries. While critical when vaccines are unavailable, host-directed therapies should complement, not replace, rapid vaccine development.

### 5.3. Non-Pharmaceutical Interventions

Non-pharmaceutical interventions (NPIs) are public health measures designed to stop the spread of disease without relying on drugs or vaccines. The most effective strategies are those that minimize illness and mortality while causing the least disruption to people’s daily lives. Choosing and implementing NPIs should be based on evidence, tailored to the situation, and consider how the specific virus spreads [282,283].

To control future outbreaks, it is crucial to rapidly identify cases and trace contacts. This means isolating and treating sick individuals, quarantining their contacts, and conducting thorough investigations. Strengthening healthcare systems—by expanding hospital capacity, securing medical supplies, and enhancing laboratory capabilities—ensures patients receive prompt and effective care. Recent reviews have discussed the effectiveness of contact tracing strategies, noting that they are effective for certain diseases in specific contexts that require robust health system governance, adequate resources, and community involvement [284,285]. This underscores that contact tracing cannot serve as a universal intervention; its success depends on alignment with epidemiological features and institutional capacity to sustain the effort.

Building upon these targeted interventions, some measures need to be adapted depending on the type of outbreak. For example, social distancing and travel restrictions are not as relevant for waterborne viral outbreaks. In this case, implementing water treatment and purification measures to ensure the safety of drinking water becomes crucial. Returning to normal life should happen gradually, based on vaccination rates and epidemiological data. During this transition, it is important to keep monitoring the situation and maintain preventive measures such as vaccination campaigns and testing. These steps help ensure that the return to normalcy lasts and reduces the risk of future outbreaks.

Evidence from COVID-19 NPIs Implementation: To control the rapid rise in COVID-19 cases, governments worldwide introduced a range of NPIs to reduce the number of infections. The analysis of NPIs effectiveness in 50 countries to contain the spread of COVID-19 was performed [286]. The study found that risk communication was the most impactful intervention, regardless of a country’s social, environmental, or other intervention strategies. Social distancing measures, such as cancelling mass gatherings and closing schools, also proved highly effective in reducing the spread of the virus. Other interventions had varying degrees of effectiveness. Resource allocation demonstrated a moderate positive impact, while case identification and contact tracing yielded mixed results, depending on the implementation timing and strategies. Travel restrictions’ effectiveness was also highly variable, suggesting careful consideration of timing and targeted regions for optimal impact. Interestingly, expanding healthcare and public health capacity did not show a strong correlation with case reduction. This might be due to the post-infection focus of healthcare and the potential for increased cross-infection in healthcare settings. Environmental measures were also the least effective. Therefore, a comprehensive approach combining various interventions was vital for effective COVID-19 control [287]. At the same time, risk communication and social distancing emerged as universally effective [288].

The COVID-19 pandemic has exposed critical and systemic limitations in infection prevention and control (IPC) programs and methodologies across healthcare settings worldwide [226,289]. Despite decades of established IPC frameworks, the pandemic revealed profound gaps in preparedness, resource allocation, adherence to standardized protocols, and surge capacity in even well-resourced health systems [289,290]. These deficiencies were compounded by the concurrent and accelerating burden of healthcare-associated infections and antimicrobial resistance, creating unprecedented strain on health systems and compromising patient safety globally [291]. It is imperative to acknowledge these limitations and their severe ramifications, as they directly demonstrated that the existing IPC infrastructure was inadequate to address emerging infectious disease threats of this magnitude [289].

These systemic failures prompted the World Health Assembly’s adoption of a comprehensive resolution on IPC at its seventy-fifth session in 2022, emphasizing urgent need for: robust, globally coordinated strategies, enhanced national capacities, sustained investment in IPC programs, and integration of pandemic preparedness into outbreak response frameworks to mitigate the impact of infectious diseases and antimicrobial resistance on public health and healthcare resilience.

## 6. Research and Development

Effective outbreak preparedness relies on research outputs as well as the presence of institutional laboratory infrastructure, including biosafety-level facilities, molecular diagnostics platforms, sequencing capabilities, trained personnel, and sustainable funding channels. These services are crucial for facilitating surveillance, diagnostics, vaccine assessment, and coordinated response efforts, especially during peak periods. In this context, research laboratories represent a core operational pillar of viral outbreak preparedness.

**Figure 5:**
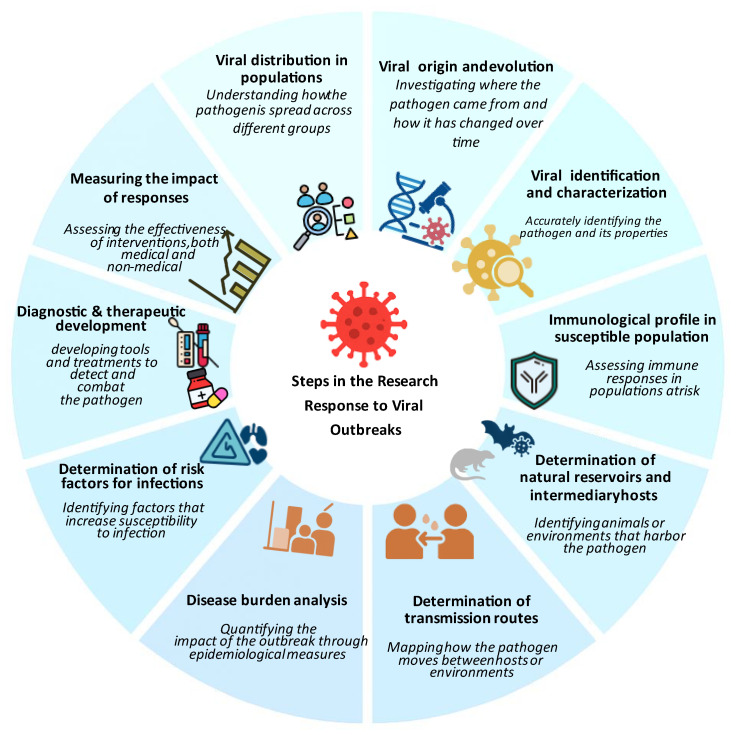
Steps in the research response to viral outbreaks. Schematic depicting major research laboratory activities during viral outbreaks, including pathogen characterization, surveillance-related analyses, and evaluation of diagnostics, therapeutics, and response measures. Icons were adapted from Flaticon (www.flaticon.com) (accessed on 1 September 2025), and the figure was created using Microsoft PowerPoint.

Although national and regional public and animal health systems take the lead in outbreak response by conducting diagnosis and detection, research laboratories contribute across all phases of outbreak preparedness [292]. Figure 5 illustrates the potential contributions made by research laboratories toward viral pandemic and epidemic preparedness. The Eastern Mediterranean Region exemplifies these contributions, which have been instrumental in preparedness and response efforts for avian influenza, MERS-CoV, and COVID-19. These laboratories built and maintained surveillance systems, conducted genetic and antigenic analyses, elucidated zoonotic reservoirs and transmission pathways, and conducted seroprevalence and vaccination efficacy investigations [293,294,295,296,297,298]. Their efforts not only informed public health officials about the actual prevalence and transmission of infectious diseases but also facilitated the development and assessment of diagnostics, medical countermeasures, and vaccines [197,298,299]. These contributions enhanced the region’s readiness and response to present and future epidemics, despite existing deficiencies in incorporating research findings into policy frameworks.

To establish an effective preparedness plan, countries should invest in research and development across various key areas:

- Understanding Viral Threats: Researching the epidemiology, transmission dynamics, and pathogenesis of viral diseases is crucial. This involves studying the genetic structure of viruses, their host range, and factors influencing their emergence and spread, with a priority focus on zoonotic viruses.
- Diagnostic Tools and Technologies: Developing new and improved diagnostic tools is essential for early detection and containment of outbreaks. This includes researching novel testing methods like POC diagnostics and multiplex assays, as well as enhancing existing laboratory-based techniques.
- Therapeutics and Vaccines: Accelerating research and development efforts for therapeutics and vaccines against viral diseases is paramount. This entails conducting preclinical and clinical trials to evaluate safety and efficacy, as well as exploring innovative approaches such as monoclonal antibodies, antiviral peptides, and RNA-based therapeutics.
- Surveillance and Monitoring Tools: Innovating in surveillance and monitoring tools enhances early detection and response to outbreaks. Research in this area focuses on developing algorithms for analyzing large datasets using artificial intelligence, such as machine learning, creating real-time monitoring systems, and integrating diverse information sources to identify potential threats and assess their impact.
- Capacity Building and Collaboration: Strengthening research capacity, infrastructure, and partnerships at national and international levels is essential. This involves supporting research institutions, academic centers, and public health laboratories and fostering collaboration among scientists, clinicians, policymakers, and industry stakeholders to accelerate progress towards shared goals.

## 7. Global Equity and Response Coordination

In addition to technical and regulatory considerations, pandemic preparedness is influenced by political, economic, and structural factors that determine global equity. COVID-19 revealed that vulnerabilities in LMICs arose not solely from technical gaps but from systemic political and structural constraints that undermined their ability to prevent, detect, and respond to emerging infectious threats [221,300]. These systemic inequities underscore the need for effective governance structures that can operationalize pandemic preparedness at both national and international levels.

Moreover, global financing remains fragmented and heavily donor-dependent. Mechanisms such as the PEF and ACT-A demonstrated limited capacity for timely, equitable funding. COVAX mobilized only a fraction of resources, while bilateral agreements allowed wealthy countries to secure disproportionate doses, perpetuating inequity [301,302,303]. Chronic underinvestment in primary care, public health institutes, laboratory networks, and surveillance systems further limited LMICs’ capacity to scale diagnostics, genomic sequencing, and vaccination programs [304].

In addition, data-sharing inequities persist despite platforms like GISAID, GISRS, and GOARN. LMICs contributed disproportionately to pathogen samples and clinical data but received limited benefit from downstream development of diagnostics, therapeutics, and vaccines [300]. Intellectual property, pricing transparency, and access to biological materials created additional barriers, weakening early-warning systems and perpetuating inequitable benefits. The WHO Pandemic Agreement (2025) aims to improve surveillance, equitable distribution, financing, and governance. However, its impact depends on enforceability and national implementation. Without binding commitments for technology transfer, regional manufacturing, and transparent allocation, inequities seen in ACT-A and COVAX may persist [300]. Structural transformation of global health governance is essential to prevent repeating COVID-19-era inequities.

Finally, addressing these political, structural, and economic barriers is critical to ensure scientific advances—flexible vaccine platforms, rapid regulatory pathways, and diagnostics—translate into equitable protection. Strengthening regional manufacturing hubs, establishing predictable financing, implementing transparent data-sharing with benefit-sharing, and aligning governance with health equity principles are essential for resilient preparedness. Without these reforms, future pandemics may reproduce the inequities observed during COVID-19, prolong outbreaks, and exacerbate global health disparities.

The requirement for swift action during viral epidemics represents a fundamental component of modern public health governance. In the past, national Ministries of Health were the main decision-makers. However, because health problems are increasingly complex, integrated, whole-of-society approaches have become necessary [305]. Recent pandemics have demonstrated that traditional health-sector-centric approaches are insufficient, highlighting the limitations of conventional governance and the need for broader institutional engagement beyond clinical professionals [306,307]. By linking global equity considerations to national decision-making, governments can better design response strategies that mitigate structural vulnerabilities.

The establishment of National Superior Committees represents a pivotal evolution in governance, marking a move from fragmented responses to cross-sectoral alignment. By structuring committees to incorporate defense, finance, and foreign affairs, governments aim to reconcile trade-offs between short-term health restrictions and long-term economic stability, demonstrating a link between governance design and outcomes [308]. While Ministerial Committees centralize decision-making to optimize resource allocation, their effectiveness depends on the integration of Emergency Operations Centers (EOCs). According to CDC [309] and Schiff & Mallinson [310], EOCs are the most important filter that keeps political expediency from overriding epidemiological data, ensuring operational decisions are informed by evidence rather than politics.

However, even well-designed governance structures face operational challenges, highlighting that centralization alone is insufficient without clear roles and communication. Analysis indicates that clearly defined roles and strong communication channels are not merely administrative details but key determinants of timely and effective responses; their absence can exacerbate infection spread [311].

To address these limitations and ensure equitable outcomes, international coordination mechanisms such as the 2025 WHO Pandemic Agreement and the Global Supply Chain and Logistics Network (GSCL) have been established. These initiatives directly address observed bottlenecks and inequities, demonstrating how institutional reforms can influence global health outcomes [312]. Furthermore, adopting the One Health approach recognizes that human health security is inseparable from animal and environmental surveillance. However, institutional frameworks alone are insufficient; the effectiveness of these systems ultimately depends on sustained political commitment and international cooperation, even when immediate crises are less pressing.

## 8. Conclusions and Future Directions

Effective surveillance, quick diagnostics, vaccine development, and treatment plans are still the most critical parts of being ready for and responding to an outbreak. To avoid delays that have made it harder to control outbreaks in the past, it is vital to strengthen traditional surveillance with digital tools and WBS, make sure that diagnostic tools are ready, and invest in adaptable vaccine and therapy platforms. NPIs are still an essential part of biomedical strategies. Long-term readiness, on the other hand, depends on ongoing collaboration across sectors, proactive research, and fair access to resources around the world. Hence, the following directions are crucial to prepare appropriately and respond to viral outbreaks.

Boost the Early Warning System:
  - Make an investment in integrated surveillance that includes WBS, real-time digital monitoring technologies, and clinical reporting.
  - Integrate One Health concepts to monitor zoonotic spillover at the interface between humans, animals, and the environment.
Increase Rapid Diagnosis:
  - Within weeks of pathogen detection, scale up low-cost, high-sensitivity POC diagnostic assays or portable devices and implement them across the country.
  - Encourage open access platforms (such as metagenomic assays) for objective, sequence-independent detection.
Increase Immunization Readiness:
  - Give top priority to the development of multiplex and prototype vaccinations that target high-risk virus families.
  - Keep critical vaccine supplies on hand and use education and awareness initiatives to overcome reluctance.
  - To shorten development durations, use flexible platforms like viral vector vaccines and mRNA.
Boost Antiviral Treatment Readiness:
  - Develop broad-spectrum antivirals and keep emergency supplies of necessary medications on hand.
  - Examine and reuse approved medications for action against newly discovered viruses in a methodical manner.
Optimize NPIs by:
  - Ensuring that health systems have the flexibility to scale personnel, hospital beds, and logistics during periods of high epidemiological demand.
  - Use open, culturally appropriate communication to encourage adherence to mask use, distance, and hygiene practices.
  - In accordance with the risk of transmission, implement strict contact tracing, isolation, and targeted travel procedures.
Encourage ongoing development:
  - Obtain specific funds for studies in implementation science, vaccinations, treatments, and diagnostics.
  - Establish official international data-sharing agreements and coordinated training programs to guarantee that readiness frameworks remain adaptable and interoperable.

Taken together, integrating these strategies into a cohesive health preparedness framework enables a shift from reactive outbreak management to proactive preparedness. Sustained commitment to innovation, equity, and global collaboration will be essential for building resilient systems capable of confronting future viral threats.

## Figures and Tables

**Table 1:** Key viral outbreaks and lessons learned for public health preparedness, 2009–2025.

Year	Pathogen	Location(s)	Key Preparedness Lessons Learned	Reference
2009	H1N1 Influenza A virus	Global	Early global surveillance enabled rapid detection; gaps in preparedness, surge capacity, and risk communication; delays and inequities in vaccine availability; need for adaptable and equitable response systems.	[7]
2010–2012	Rift Valley fever virus	South Africa, Mauritania	Need for integrated One Health surveillance; importance of climate and vector monitoring to anticipate outbreaks.	[8]
2010–2014	Chikungunya virus	Indian Ocean islands, Asia, spread to Americas	Importance of vector surveillance and rapid detection; global travel accelerates spread; need for community mosquito-control strategies.	[9]
2012–ongoing	MERS-CoV	Middle East (especially Saudi Arabia), exported cases globally	Importance of real-time genomic surveillance; nosocomial transmission control; zoonotic reservoir monitoring (camels).	[10]
2013	H7N9 avian influenza	China	Early detection in live poultry markets; need for sustained genomic surveillance; importance of risk communication and cross-sectoral coordination.	[11]
2013	H1N1 seasonal influenza waves, “post-pandemic H1N1 seasonal waves (2010s)”	Global	Reinforced need for strong vaccine manufacturing pipelines and annual preparedness; importance of surveillance for antigenic drift.	[12]
2013–2014	Polio resurgence	Syria, Horn of Africa	Impact of conflict on vaccination systems; need for emergency immunization campaigns and global coordination.	[13]
2014–2016	Ebola virus (Zaire ebolavirus)	West Africa (Guinea, Sierra Leone, Liberia)	Weak health systems amplify outbreaks; need for rapid diagnostics, community engagement, and global surge capacity.	[14]
2015–2016	Zika virus	Americas (Brazil, Colombia)	Integration of vector control, reproductive health services, and real-time surveillance; addressing asymptomatic transmission.	[15]
2018–2020	Ebola virus (DRC outbreak)	DRC	Genomic surveillance (MinION), ring vaccination, field sequencing; logistics in conflict zones critically affect outcomes.	[16]
2019–2020	Measles resurgences	Samoa, DRC, Ukraine	Declining vaccination coverage drives explosive outbreaks; importance of routine immunization + misinformation management.	[17]
2020–present (PHEIC 2020–May 2023)	SARS-CoV-2 (COVID-19)	Global	Need for scalable genomic surveillance, supply chain resilience, rapid vaccine platforms, and equitable access to countermeasures.	[18]
2022	Mpox (Clade IIb)	Europe, Americas, global spread	Early detection in non-endemic regions; sexual-network surveillance; potential of WBS.	[19]
2024	Dengue virus (record global cases)	Americas, Southeast Asia	Climate-driven vector expansion; need for climate-linked forecasting and integrated vector management.	[20]
2024–2025	H5N1 avian influenza (B3.13 genotype)	USA (dairy cattle), global birds	Urgent need for One Health surveillance; cross-species genomic monitoring; bulk milk testing enabled early detection.	[21]
2025	Sudan ebolavirus	Uganda	Decentralized sequencing enabled <24 h confirmation; vaccine gaps for non-Zaire Ebola species remain a major preparedness issue.	[22]

**Table 3:** Key surveillance tools across domains and their corresponding gaps in coverage, capacity, and integration.

Surveillance Domain	Existing Tools/Networks	Key Gaps/Challenges
**Case-based (human)**	National notification systems (IHR reporting, IDSR), sentinel sites (flu, SARI), NNDSS, and TESSy.	Many countries under-report cases; coverage gaps in rural/LMIC settings; delays in data sharing [134]. Labs may be limited, leading to slow confirmation.
**Event-based (early warning)**	Global alert systems (WHO EIOS), platforms (ProMED, HealthMap, Nextstrain alerts), collaborative surveillance frameworks (WHO Hub for Pandemic and Epidemic Intelligence, 2024).	Data are unstructured and sometimes false; alerts often need follow-up confirmation. Coverage is patchy in low-access regions. Integration into official surveillance is limited [135].
**Genomic sequencing**	GISAID and country genomics networks (e.g., national influenza centers, COVID Genome initiatives). Emerging: Real-time surveillance systems (UK Respiratory Metagenomics Programme, 2024).	Sequencing capacity and bioinformatics vary widely [136]. Many regions still cannot sequence routinely. Data sharing lags and is uneven, slowing variant tracking [62].
**Animal/One-Health**	OIE (WOAH) animal disease reporting, FAO/WHO zoonoses networks; sentinel livestock/wildlife programs. Quadripartite One Health Joint Plan of Action (FAO, WOAH, UNEP, WHO, 2022–2026).	Human–animal surveillance is poorly integrated. Wildlife and farm surveillance are limited. Many zoonotic hotspots have no routine animal testing, delaying detection of spillovers [134].
**Environmental surveillance**	Wastewater monitoring (used for PV, expanded for SARS-CoV-2); vector surveillance (mosquito trapping for arboviruses).	Coverage is limited to areas with sanitation infrastructure (Crone & Freemont, 2024). Few data from rural or informal settlements. Environmental methods are still pilot-stage and not globally standardized.
**Data sharing & analytics**	Platforms like WHO’s Global Health Observatory, CDC Epicenters, DHIS2 (in some countries). WHO Hub for Pandemic and Epidemic Intelligence; BRIDGE Alliance	Systems are siloed by country/sector; lack real-time interoperability. During outbreaks, slow data reporting and privacy/legal issues hinder global situational awareness.

**Table 4:** Technology readiness of diagnostics and vaccines by major viral category. “TRL ≈ 9” indicates widely deployed tools (e.g., PCR tests or licensed vaccines), lower values indicate earlier stages.

Viral Category (Examples)	Diagnostics (Readiness)	Vaccines (Readiness)
Respiratory viruses (SARS-CoV-2, Influenza, RSV, Measles)	Widespread PCR and rapid tests (TRL ≈ 9) exist for flu and SARS-CoV-2. RSV/flu point-of-care tests are common; measles lab tests are routine.	Licensed vaccines for influenza (annual), measles (MMR) and now RSV (first approved 2023) [149] (TRL ≈ 9). SARS-CoV-2 vaccines (mRNA, vector) are TRL ≈ 9.
Filoviruses & Hemorrhagic (Ebola, Marburg, Lassa)	PCR and some rapid assays exist (Ebola RDTs in TRL 7–8), but coverage is limited. Early detection often delayed [136]. Lassa diagnostics are mainly PCR (TRL ≈ 6).	Ebola vaccine (rVSV-Zebov) is licensed (TRL ≈ 9), Marburg and Lassa vaccines are in development (TRL ≈ 4–7) with few in human trials.
Henipaviruses (Nipah, Hendra)	PCR tests are available (TRL ≈ 6–7) but not widely deployed; field assays are experimental.	No licensed vaccines; candidates (vector or subunit) are in early/animal stages (TRL ≈ 3–5).
Arboviruses (Dengue, Zika, Chikungunya, West Nile)	PCR and serologic tests exist for dengue and Zika (TRL ≈ 7–8), but antigen tests suffer cross-reactivity. Chikungunya PCR available.	Dengue: one tetravalent vaccine licensed (TRL ≈ 8, limited use). Zika, chikungunya, WNV: no licenced vaccines (TRL ≈ 3–6; several candidates in trials).
Other (e.g., PV, HIV) (Enteroviruses/Retroviruses)	PV: sensitive lab diagnostics/AFP surveillance exist (TRL ≈ 9). HIV: high-quality PCR/ELISA tests (TRL ≈ 9).	PV vaccines (OPV/IPV) were TRL ≈ 9 (nearly eradicated). HIV: despite decades of effort, no effective vaccine (TRL ≈ 2–3).

**Table 5:** The challenges to diagnostic preparedness and potential solutions.

	Challenges	Proposed Solutions
**Research and Development**	Lack of a diagnostic for field use	Develop rapid, simple diagnostic platforms with minimal sample prep and training needs.
	Insufficient funding and donor coordination cause duplication of efforts	Set up a central body to coordinate and oversee funding for diagnostics. Connect small start-ups or academic groups with larger diagnostic, vaccine, or pharmaceutical manufacturers with greater production capacity.
	Low commercial sustainability of diagnostics outside outbreak periods	Offer market incentives to manufacturers and develop sustainable business models to balance losses during non-outbreak periods. Provide funding for the stockpiling of tests.
	Limited sample availability slows down progress in diagnostic development	Establish a specimen sample bank, with defined storage sites and clear procedures for access.
	Limited collaboration between experts and laboratories with pathogen-specific expertise	Expand expert personnel and laboratory networks to allow more rapid responses during outbreaks and maximize knowledge sharing. Partner with diagnostics and vaccine developers to find novel diagnostic targets.
	Delays in the sharing of diagnostic data are affecting response and containment times	Create connectivity solutions enabling real-time data reporting.
**Logistical and healthcare system**	Shortages of diagnostic materials and supply chain interruptions during outbreaks	Preselect suppliers to ensure appropriate capacity for outbreak situations. Establish manufacturing lines for diagnostic production during outbreaks.
	Poor diagnostic and surveillance capacity at the national level in many countries	Strengthen country-specific surveillance lab networks for routine testing. Train healthcare workers in real-time reporting. Integrate diagnostics and vaccines into unified health programs. Implement One Health surveillance across human, animal, and environmental sectors.

## Data Availability

No new data were created or analyzed in this study. Data sharing is not applicable to this article.

## Abbreviations

The following abbreviations are used in this manuscript:

CDC	Centers for Disease Control and Prevention
CMV	Cytomegalovirus
COBRA	Computational Optimized Broadly Reactive Antigen
CRISPR	Clustered Regularly Interspaced Short Palindromic Repeats
ddPCR	Droplet Digital PCR
EIOS	Epidemic Intelligence from Open Sources
ELISA	Enzyme-Linked Immunosorbent Assay
EOCs	Emergency Operations Centers
FDA	US Food and Drug Administration
GDD	Global Disease Detection
GISAID	Global Initiative on Sharing All Influenza Data
GISRS	Global Influenza Surveillance and Response System
GPHIN	Global Public Health Intelligence Network
gRNAs	Guide RNAs
HEIs	Higher Education Institutions
HSV	Herpes Simplex Virus
IHR	International Health Regulations
JAK	Janus kinase
LFA	Lateral Flow Assay
LMICs	Low- and Middle-Income Countries
LNP-mRNA	Lipid Nanoparticle mRNA
mAbs	Monoclonal Antibodies
MERS	Middle East Respiratory Syndrome
MoHP	Ministry of Health and Population
MPXV	Monkeypox Virus
NGS	Next-Generation Sequencing
NPIs	Non-pharmaceutical Interventions
PHEIC	Public Health Emergency of International Concern
POC	Point-of-Care
ProMED	Program for Monitoring Emerging Diseases
PV	Poliovirus
RNA	Ribonucleic Acid
RT-LAMP	Reverse Transcription Loop-Mediated Isothermal Amplification
RT–qPCR	Quantitative Reverse Transcription Polymerase Chain Reaction
SARS-CoV	Severe Acute Respiratory Syndrome Coronavirus
WBS	Wastewater-Based Surveillance
